# Association between socioeconomic status and survival in patients with hepatocellular carcinoma

**DOI:** 10.1002/cam4.4223

**Published:** 2021-08-20

**Authors:** Yongshun Zheng, Xun Zhang, Jinsen Lu, Shuchen Liu, Yeben Qian

**Affiliations:** ^1^ Department of General Surgery The First Affiliated Hospital of Anhui Medical University Hefei Anhui China; ^2^ Department of Orthopedics Affiliated Anhui Provincial Hospital of Anhui Medical University Hefei Anhui China

**Keywords:** hepatocellular carcinoma, nomograms, SEER program, socioeconomic factors

## Abstract

**Background:**

The effect of socioeconomic status (SES) on hepatocellular carcinoma (HCC) is still unclear, and there is no nomogram integrated SES and clinicopathological factors to predict the prognosis of HCC. This research aims to confirm the effects of SES on predicting patients’ survival and to establish a nomogram to predict the prognosis of HCC.

**Methods:**

The data of HCC patients were collected from the Surveillance, Epidemiology, and Final Results (SEER) database from 2011 to 2015. SES (age at diagnosis, race and sex, median family income, education level, insurance status, marital status, residence, cost of living index, poverty rate) and clinicopathological factors were included in univariate and multivariate Cox regression analysis. Nomograms for predicting 1‐, 3‐, and 5‐year cancer‐specific survival (CSS) and overall survival (OS) were established and evaluated by the concordance index (C‐index), the receiver operating characteristic curve (ROC), the calibration plot, the integrated discrimination improvement (IDI), and the net reclassification improvement (NRI).

**Results:**

A total of 33,670 diagnosed HCC patients were involved, and nomograms consisting of 19 variables were established. The C‐indexes of the nomograms are higher than TNM staging system, which predicts the CSS (0.789 vs. 0.692, *p* < 0.01) and OS (0.777 vs. 0.675, *p* < 0.01). The ROC curve, calibration diagram, IDI, and NRI showed the improved prognostic value in 1‐, 3‐, and 5‐year survival rates.

**Conclusion:**

SES plays an important role in the prognosis of HCC patients. Therefore, policymakers can make more precise and socially approved policies to improve HCC patients’ CSS and OS.

## Graphical abstract

The impacts of socioeconomic status (SES), including: age at diagnosis, race and sex, median family income, education level, insurance status, marital status, residence, cost of living index, poverty rate on hepatocellular carcinoma (HCC) are still unclear. This research collected the data of 33,670 diagnosed HCC patients from the Surveillance, Epidemiology, and Final Results (SEER) database, and nomograms for 1‐, 3‐, and 5‐year survival rates based on the SES and clinical factors were constructed.

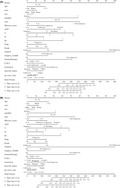

## 1 INTRODUCTION

Hepatocellular carcinoma (HCC) is the most common type of primary liver cancer, the sixth most common cancer and the third to fourth most deadly cancer.^1^, ^2^ Due to the lack of specific clinical manifestations, HCC is often detected at the intermediate‐to‐advanced stage.^3^ In the United States, the death rate from HCC increased by 43% (from 7.2 to 10.3 deaths per 100,000) between 2000 and 2016 with a 5‐year survival of 18%.^4^, ^5^ The clinical practice commonly uses the American Joint Committee on Cancer (AJCC) TNM staging system and the National Comprehensive Cancer Network guidelines to predict patient prognosis.^6^ Nomograms, developed based on these systems and guidelines, are a more reliable model for statistical prediction. It can be used to predict individual survival in conjunction with risk factors in tumor development, allowing it to be used to identify and stratify patients.^7^ However, most of the existing clinical prediction models have only clinicopathological factors, including tumor size, alpha fetoprotein (AFP), tumor stage, etc.^8^ The impacts of patients’ socioeconomic status (SES) on HCC prognosis are often overlooked. In fact, their survival outcomes usually change when patients with different SESs receive the same or various treatment. These SESs that may affect patient prognosis are not included in the prediction model, making the result less accurate. Moreover, sociodemographic factors, such as age at diagnosis, race, and sex, are closely related to SES, so this research mainly analyzed the relationship between these factors and SES.^9^ Meanwhile, it is confirmed that marital condition could influence the HCC prognosis since patients can receive caring and emotional support from partners.^10^, ^11^ Also, it was reported that income and insurance status might affect the diagnosis of the disease and compliance with subsequent treatment.^12^, ^13^ It is because there was a significant correlation between patients with high income or medical insurance and higher treatment uptake, which in turn was associated with survival.^12^, ^13^ The developmental degree of the patients’ places of residence and the poverty rate at the county level were related to access to medical resources, while their education level might affect their compliance with follow‐up treatment.^14^ Due to the intrinsic relevance of SES, we included both cost‐of‐living index (COLI), which are meant to estimate the expenses an average person needed to acquire food, housing, transportation, health care, child care, other necessities, and taxes in each state (metropolitan and non‐metropolitan).^15^ The index value is the ratio of the local cost‐of‐living to the US population‐weighted mean cost‐of‐living.^16^ Counties with values over 1.0 have a higher cost‐of‐living than the US mean, and counties with values <1.0 have lower cost‐of‐living.^17^

The Surveillance, Epidemiology, and End Results (SEER) Program is a critical population‐based database, a definitive source of information on cancer in the United States. This database includes 18 population‐based cancer registries and covers 30% of the United States population.^18^ In the case of poverty, education and other SESs, the population covered by SEER can represent the general US population.^19^ The related information of patients taken from the dataset is more likely to be generalizable in constructing nomograms.^7^ This article extracted the data from SEER and aimed to identify the impacts of SES on HCC patients and create nomograms separately based on cancer‐specific survival (CSS) and overall survival (OS), improving the accuracy of nomograms in predicting HCC patients’ prognosis.

## 2 MATERIALS AND METHODS

### 2.1 Data source and selection

This paper extracted the data of diagnosed HCC patients from SEER (1975–2016), and Official SEER*Stat software (Version 8.3.8; NCI, Bethesda, MD, USA) was used to collect data. The SEER dataset would not provide case identification information, so using these data does not require patients’ consent. We included data on patients diagnosed with HCC from 2011 to 2015, including eligible cases according to the following criteria: (i) at the time of diagnosis, the patient had only primary liver cancer based on ICD‐O‐3 (Third Edition of the International Classification of Diseases for Oncology) and the primary location was in the liver rather than the intrahepatic bile duct. (ii) patients with unknown survival months, vital status, cancer causes of death, TNM staging system, race, and residence were excluded.

### 2.2 Variables

**FIGURE 1:**
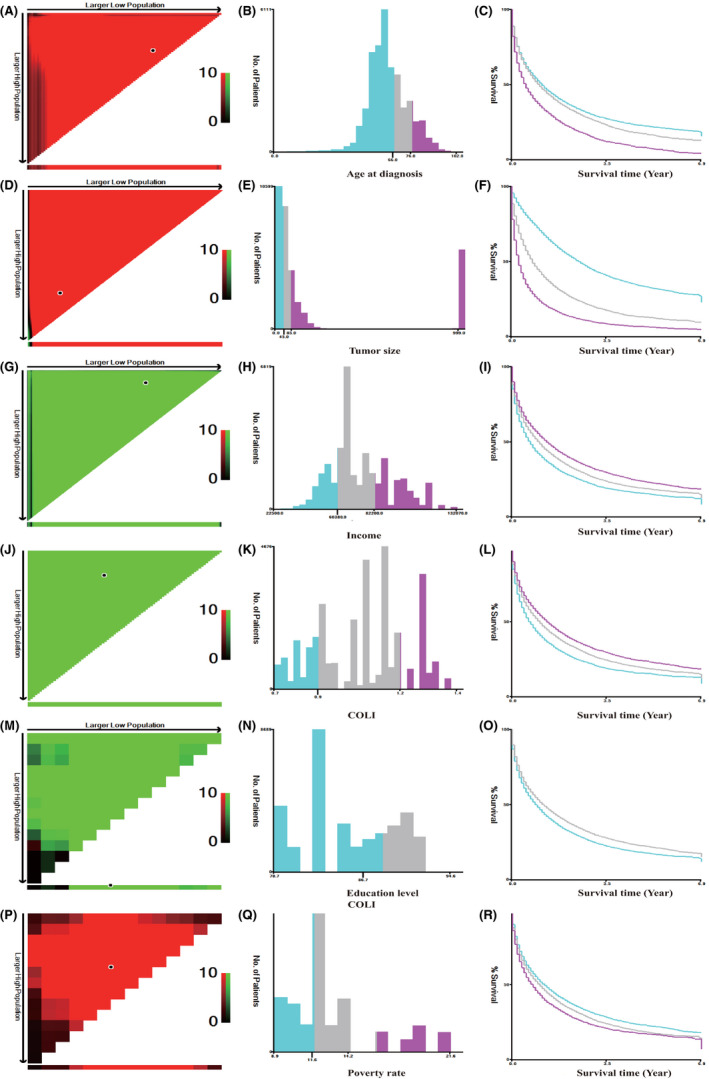
X‐tile analysis. (A–R) The best cut‐off points of age at diagnosis, income, COLI, education level, and poverty rate were defined via the X‐tile program. (A, D, G, J, M, P) The black dot indicates that optimal cutoff values have been identified. (B, E, H, K, N, Q) A histogram and (C, F, I, L, O, R) Kaplan–Meier were constructed based on the cut‐off points. COLI, cost‐of‐living index

Variables that were involved in the research include SES (age at diagnosis, race, sex, median family income, education level, insurance status, marital status, residence, COLI, poverty rate), clinicopathological factors (primary tumor number, tumor size, AFP, Fibrosis Score, the 7th edition of AJCC TNM staging system, metastasis to bone, metastasis to brain, metastasis to lung, regional lymph nodes removed for examination, regional nodes surgery, surgery, chemotherapy, radiotherapy). Patients with TX or NX of TNM staging system, or unknown of metastasis to brain, lung or bone were included, since the data of these variables were unable to assess rather than unknown. For example: half of the TX patients were N0 or N1 and patients with unknown of metastasis to brain may have metastasis to bone or lung and vice versa. Age, size, income, COLI, education level, and poverty rate were categorized based on X‐tile program (Yale University, New Haven, CT, USA) to get the best cut‐off points (Figure 1). Education level represents the ratio of patients who had high school graduate or higher at the age of 25 or more, and education level and poverty rate were county level, instead of individual level. The research’s result was HCC CSS and OS. CSS refers to the date of diagnosis to the date of death due to HCC, while OS is defined as the time from the date of diagnosis to the date of death due to unlimited reasons. The date of the last follow‐up visit is December 31, 2015.

### 2.3 Statistical analysis

All statistical analyses were utilized R software version 4.03 (http://www.r‐project.org/). Also, the rms, foreign, survival, survivalROC, caret, survC1 and survIDINRI packages were used in R software. The Kaplan–Meier (KM) method and Log‐rank tests were operated to create the cumulative survival curve and determine the prognostic factors. Independent risk factors could be identified through multivariate Cox regression analysis. The stepwise regression was used for controlling potential confounders, which will lead to multicollinearity.^20^ Two prognostic nomograms were constructed according to the results of multivariate analysis to predict the OS and CSS for 1‐, 3‐, and 5‐ years. Among the factors in nomogram, the highest score is 100 points. So patients calculated the total scores based on each factor’s scores and a line is drawn downward to the survival axes to obtain the 1‐, 3‐, and 5‐year survival rates. The performance of the nomogram were evaluated via the concordance index (C‐index), receiver operating characteristic (ROC) curve, and area under the curve (AUC). The calibration curves were subjected to 1000 bootstraps resamples to assess the actual survival against the nomogram‐predicted probabilities. Besides, by calculating integrated discrimination improvement (IDI) and the net reclassification improvement (NRI), this research compared the prediction value of nomograms and TNM staging system. Meanwhile, we used IDI and NRI to compare the differences of nomograms between with and without socioeconomic factors. The statistical significance was defined as *p* < 0.05 of the two‐sided tests.

## 3 RESULTS

### 3.1 Patient characteristics

According to the inclusion criteria, a total of 43,321 HCC patients were extracted from the SEER database, 9651 were excluded according to the exclusion criteria, and finally 33,670 patients were included in the study. The included data were randomly assigned to the training cohort (*n* = 23,570) and the validation cohort (*n* = 10,100). The main SES of patients was ≤66 years old, White race, male, COLI between 0.885 and 1.167, income between 60,460 and 82,200 ($), private insured, marital status married or domestic partner, living in the metropolitan. The poverty rate and education level at the county level were 1.8%–14.2% and 78.7%–86.7%, respectively. The baseline characteristics of training set are shown in Table 1.

**TABLE 1:** Multivariate Cox analysis of the training set on CSS and OS

Variables	Patient no. (%)	CSS	OS
		HR (95% CI)	HR (95% CI)
Age (years)			
≤66	1579 (67.0)	Reference	Reference
67–76	4797 (20.4)	1.17 (1.12–1.22)***	1.15 (1.11–1.20)***
≥77	2977 (12.6)	1.30 (1.24–1.37)***	1.34 (1.28–1.40)***
Race			
White	16,389 (69.5)	Reference	Reference
Black	3305 (14.0)	0.95 (0.91–1.00)*	0.98 (0.93–1.02)
Other	3876 (16.5)	0.87 (0.83–0.92)***	0.85 (0.82–0.89)***
Sex			
Male	18,311 (77.7)	Reference	Reference
Female	5259 (22.3)	0.92 (0.88–0.96)***	0.91 (0.87–0.94)***
Primary tumor number			
1	22,781 (96.7)	Reference	Reference
≥2	789 (3.3)	0.48 (0.43–0.54)***	0.67 (0.61–0.74)***
Tumor size (mm)			
≤45	10,303 (43.7)	Reference	Reference
46–85	5182 (22.0)	1.55 (1.47–1.63)***	1.44 (1.37–1.50)***
≥86/unknown	8085 (34.3)	2.14 (2.03–2.25)***	1.94 (1.85–2.04)***
Fibrosis score			
0–4	1087 (4.6)	Reference	Reference
5–6	5575 (23.7)	1.16 (1.06–1.28)**	1.21 (1.11–1.33)***
Unknown	16,908 (71.7)	1.28 (1.17–1.40)***	1.32(1.21–1.43)***
AFP			
Positive	13,435 (57.0)	Reference	Reference
Negative	4711 (20.0)	0.62 (0.59–0.65)***	0.66 (0.63–0.69)***
Bordline	37 (0.2)	0.92 (0.60–1.41)	0.87 (0.58–1.31)
Unknown	5387 (22.8)	0.78 (0.74–0.81)***	0.79 (0.76–0.82)***
T			
0/1	9396 (39.9)	Reference	Reference
2	4725 (20.0)	1.39 (1.32–1.46)***	1.29 (1.23–1.35)***
3	5341 (22.7)	1.72 (1.64–1.81)***	1.60 (1.53–1.68)***
4	855 (3.6)	1.96 (1.80–2.13)***	1.80 (1.66–1.95)***
X	3253 (13.8)	1.32 (1.24–1.42)***	1.27 (1.19–1.35)***
N			
0	18,909 (80.2)	Reference	Reference
1	1642 (7.0)	1.27 (1.20–1.35)***	1.26 (1.19–1.33)***
X	3019 (12.8)	1.15 (1.07–1.23)***	1.14 (1.07–1.22)***
M			
0	20,334 (86.3)	Reference	Reference
1	3236 (13.7)	1.62 (1.52–1.73)***	1.57 (1.48–1.67)***
Metastasis to brain			
Yes	75 (0.3)	Reference	Reference
No	21,792 (92.5)	0.90 (0.70–1.15)	0.91 (0.72–1.15)
Unknown	1703 (7.2)	0.93 (0.68–1.23)	0.92 (0.67–1.25)
Metastasis to lung			
Yes	1299 (5.5)	Reference	Reference
No	20,533 (87.1)	0.74 (0.69–0.80)***	0.75 (0.70–0.81)***
Unknown	1738 (7.4)	0.93 (0.78–1.10)	0.91 (0.77–1.08)
Metastasis to bone			
Yes	955 (4.1)	Reference	Reference
No	20,928 (88.8)	0.94 (0.86–1.02)	0.97 (0.89–1.05)
Unknown	1687 (7.1)	0.84 (0.68–1.04)	0.88 (0.72–1.08)
Node			
No	21,043 (89.3)	Reference	Reference
Yes	827 (3.5)	0.86 (0.73–1.01)	0.85 (0.73–0.98)*
Unknown	1700 (7.2)	0.74 (0.68–0.81)***	0.76 (0.70–0.83)***
Surgery			
Yes	5301 (22.5)	Reference	Reference
No/unknown	18,269 (77.5)	3.57 (3.37–3.78)***	3.40 (3.23–3.58)***
Surgery_lymph			
Yes	659 (2.8)	Reference	Reference
No/unknown	22,911 (97.2)	1.34 (1.09–1.65)**	1.29 (1.06–1.55)**
Chemotherapy			
Yes	9744 (41.3)	Reference	Reference
No/unknown	13,826 (58.7)	1.92 (1.85–1.99)***	1.95 (1.88–2.01)***
Cost‐of‐living index			
≤0.882	4822 (20.4)	Reference	Reference
0.885–1.167	12,652 (53.7)	0.86 (0.82–0.91)***	0.87 (0.83–0.92)***
≥1.169	6096 (25.9)	0.78 (0.74–0.85)***	0.80 (0.75–0.86)***
Income ($)			
22,500–60,380	4896 (20.8)	Reference	Reference
60,460–82,200	11,526 (48.9)	0.99 (0.94–1.05)	0.98 (0.94–1.03)
82,940–132,070	7148 (30.3)	1.01 (0.94–1.09)	1.02 (0.95–1.09)
Insurance			
Medicaid	5890 (25.0)	Reference	Reference
Private insured	15,835 (67.2)	0.93 (0.89–0.96)***	0.89 (0.86–0.93)***
No/unknown	1845 (7.8)	1.22 (1.14–1.30)***	1.16 (1.09–1.23)***
Marital			
Married/domestic partner	11,359 (48.2)	Reference	Reference
Other	5507 (23.4)	1.12 (1.07–1.16)***	1.12 (1.08–1.17)***
Single	5372 (22.8)	1.07 (1.03–1.12)**	1.10 (1.06–1.15)***
Unknown	1332 (5.6)	0.89 (0.82–0.96)**	0.90 (0.84–0.96)**
Residence			
Metropolitan	21,255 (90.2)	Reference	Reference
Non‐metropolitan	2315 (9.8)	0.99 (0.93–1.05)	0.99 (0.93–1.04)
Poverty rate			
8.9%–11.6%	6897 (29.3)	Reference	Reference
11.8%–14.2%	11,798 (50.1)	1.00 (0.93–1.07)	1.00 (0.94–1.07)
16.3%–21.6%	4875 (20.6)	1.12 (1.03–1.21)**	1.11 (1.04–1.20)**
Education level			
78.7%–86.7%	14,464 (61.4)	Reference	Reference
88.5%–94.6%	9106 (38.6)	0.98 (0.92–1.05)	0.98 (0.93–1.05)

Fibrosis score: AJCC classifies fibrosis scores 0–4: none to moderate fibrosis; 5–6: severe fibrosis or cirrhosis. Fibrosis score is also called Ishak score.

Node: lymph nodes removed for examination to derive the staging basis for the N category in the TNM system.

Surgery_lymph: surgery for regional lymph node.

Education level represents the percentage of patients aged ≥25 years with at least a high school diploma. The education level and poverty rate were determined at the county‐level.

Abbreviations: AFP, alpha fetoprotein; AJCC, American Joint Committee on Cancer;CI, confidence interval; CSS, cause‐specific survival; HR, hazard ratio; OS, overall survival; T, N, M, TNM staging system (T, tumor, N, node, M, metastasis).

* *p* < 0.05.

** *p* < 0.01.

*** *p* < 0.001.

### 3.2 Survival analysis

The KM method was used to calculate the specific survival curve of HCC and the results are shown in Figure S1. Since there was no statistically significant difference in prognosis between tumor size ≥86 (mm) and tumor size unknown, they were grouped into one group. Radiotherapy was identified not associated with the significant differences in survival. Multivariate analyses for the rest variables demonstrated that income, education level, residence, metastasis to brain, and metastasis to bone were not associated with the significant differences in survival. The results of the multivariate Cox analyses of CSS and OS were listed in Table 1. The independent variables identified by the stepwise regression were consistent with the multivariate Cox analyses, which ensuring that all the independent variables were significant and to eliminate the effects of multicollinearity.^21^

### 3.3 Nomogram construction and performance

**FIGURE 2:**
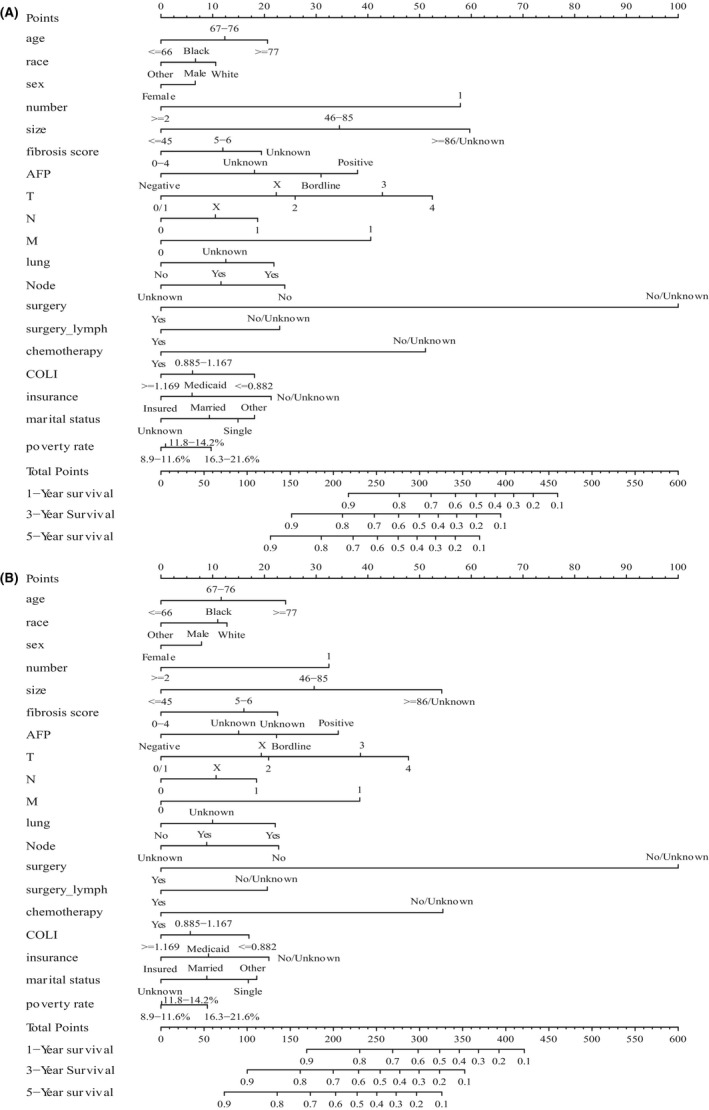
Nomograms for predicting the 1‐, 3‐, and 5‐year survival of HCC patients. (A) Nomogram based on CSS. (B) Nomogram based on OS. CSS, cancer‐specific survival; HCC, hepatocellular carcinoma; OS, overall survival

According to the risk factors obtained from multivariate Cox analyses, nomograms of predicting the HCC of the 1‐, 3‐, and 5‐year CSS and OS (Figure 2) indicated that clinicopathological factors were major impacts on patient prognosis. For example, surgery had greatest influence to the patient prognosis, followed by chemotherapy, size and TNM staging system, while SES plays a complementary role. However, the results from IDI and NRI showed that with or without socioeconomic factors significantly affect the prediction of nomograms for CSS and OS (Table S1).

**FIGURE 3:**
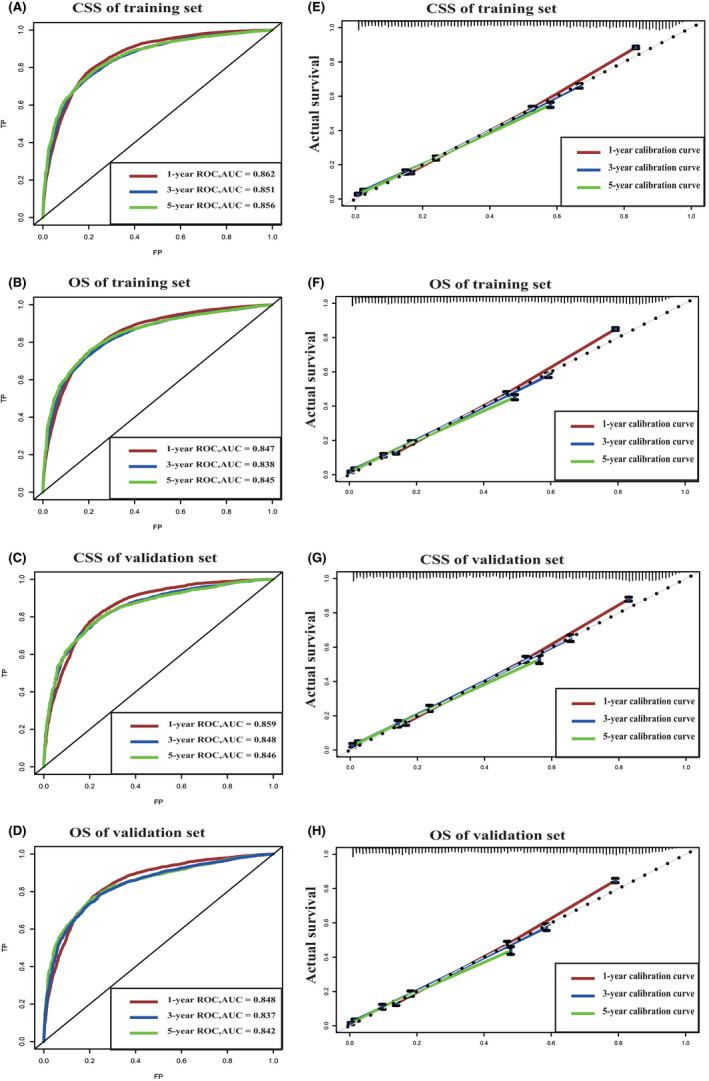
ROC and calibration curves. (A, B) show the ROC curves of the training set of 1‐, 3‐ and 5‐year survival based on CSS and OS, respectively. (C, D) show the ROC curves of the validation set of 1‐, 3‐ and 5‐year survival based on CSS and OS, respectively. (E, F) show the calibration curves of the training set of 1‐, 3‐ and 5‐year survival based on CSS and OS, respectively. (G, H) show the calibration curves of the validation set of 1‐, 3‐ and 5‐year survival based on CSS and OS, respectively. CSS, cancer‐specific survival; OS, overall survival; ROC, receiver operating characteristic curve

The C‐indexes provided by nomograms of CSS and OS were higher than TNM staging system (0.789 vs. 0.692, <0.001; 0.777 vs. 0.672, <0.001). This indicated that compared with TNM staging system, our models had better accuracy in predicting the prognosis of the HCC. The AUC of the 1‐, 3‐, and 5‐year CSS and OS of training cohorts were 0.862, 0.851, 0.856 versus 0.847, 0.838, 0.845 while the AUC of the validation cohorts were 0.859, 0.848, 0.846 vs. 0.848, 0.837, 0.842, respectively (Figure 3A–D). Calibration curves in Figure 3E–H revealed the consistency of the nomogram between predicted and actual observed 1‐, 3‐, and 5‐year CSS and OS, and depicted high consistency of the nomograms both in training and validation cohorts. The outcomes from IDI and NRI demonstrated that compared with TNM staging system, this research's nomograms had higher predictive power for CSS and OS in HCC patients (Table 2).

**TABLE 2:** IDI and NRI of the nomograms on CSS and OS

	Survival time	Items	Training set	Validation set
			Est (95% CI)	Est (95% CI)
CSS	1‐year	IDI	0.18 (0.17–0.18)***	0.17 (0.16–0.19)***
		NRI	0.47 (0.45–0.49)***	0.47 (0.45–0.50)***
	3‐year	IDI	0.20 (0.19–0.21)***	0.19 (0.18–0.21)***
		NRI	0.48 (0.47–0.50)***	0.48 (0.45–0.51)***
	5‐year	IDI	0.23 (0.22–0.25)***	0.21 (0.19–0.24)***
		NRI	0.51 (0.49–0.54)***	0.48(0.44–0.53)***
OS	1‐year	IDI	0.18 (0.17–0.18)***	0.18 (0.16–0.19)***
		NRI	0.46 (0.44–0.47)***	0.46 (0.44–0.48)***
	3‐year	IDI	0.20 (0.19–0.21)***	0.19 (0.18–0.21)***
		NRI	0.47 (0.45–0.49)***	0.46 (0.43–0.50)***
	5‐year	IDI	0.22 (0.21–0.24)***	0.21 (0.18–0.23)***
		NRI	0.49 (0.45–0.52)***	0.46 (0.33–0.51)***

Abbreviations: CI: confidence interval; CSS, cause‐specific survival; Est, Empower Stat; IDI, integrated discrimination improvement index; NRI, category‐less net reclassification index; OS, overall survival.

*** *p* < 0.001.

## 4 DISCUSSION

Similar to other cancers, the differences in HCC patients’ SES would lead to different survival outcomes, and socioeconomic disparities in cancer varied between countries and rising concern worldwide.^22^ Although the economic and medical levels vary from country to country, the results showed that low SES in cancer patients is associated with cancer‐related symptoms, low quality of life, and a short survival periods.^23^, ^24^, ^25^ To our knowledge, this is the first attempt to include SES to construct nomograms for CSS and OS to predict the prognosis of HCC patients. The present study provided important information to assist the development of the national cancer policy and support the low SES of HCC patients, thereby improving the prognosis of patients.

The data the study used were representative because they were extracted from the SEER database, which contains reliable information, a wide range of patient sources, and large sample size. Adding SES and clinicopathological factors, rather than solely studying them, decreased confounding variables. It is because there are some connections between different factors, which might impact the results of prognosis. Previous studies had reported that age, race, sex, marital status, insurance, income, residence, and education level might influence the prognosis of cancer patients.^10^, ^26^, ^27^, ^28^, ^29^, ^30^ Based on this, our study included two more factors, COLI and poverty. As the smallest geographic unit in policy legislations, it is reasonable to assess individual indirectly based on the county‐level variables and previous studies adopted both individual variables and county‐level variables to construct the nomograms.^31^, ^32^ Obviously, older age was related to poor prognosis for HCC patients, since older patients often have more comorbidities, including cardiovascular diseases and metabolic disorders, and worsened at the time of diagnosis due to lack of monitoring, which was considered not cost‐effective in patients without advanced fibrosis/cirrhosis.^33^, ^34^ On the other hand, patients with HCC experienced poor quality of life. Approximately half of HCC patients in the USA do not undergo any treatment, and most of them are older age, African American race, and no insurance because their income level is low.^35^, ^36^, ^37^ The median survival time of these untreated patients were 13.4, 9.5, 3.4, and 1.6 months depend on the TNM stages 0/1, 2, 3, and 4, respectively.^38^ With the development of medical techniques, the survival rate of patients with HCC increased in general, but it was not uniform with respect to race with worse survival in African Americans and superior in Asian population.^39^, ^40^ On one side, this phenomenon may be related to the genetic susceptibility; for example, HCC in Asians is mostly associated with hepatitis B virus infection and the related treatment has been gradually improved.^41^ On the other side, it may correlate with separate and unequal systems of health care systems, clinicians constrain the resources, and reinforce implicit bias.^42^ Additionally, income inequality across racial groups is common in the United States. In particular, Blacks, American Indians, and Hispanics have lowest income, exacerbating the inequality of medical services.^43^ Similar to race, the effect of sex on prognosis was also shown by genetic susceptibility and SES. Overall, in this research, the number of HCC cases was over threefold higher in men than in women. Hormones and reproductive factors can reduce the HCC risk since estrogen is considered as a protective factor, while testosterone may promote tumorigenesis.^44^ However, in older patients, the incidence of HCC remains more than three times higher in men than in menopausal women, probably because men have higher rates of hepatitis C virus infection and alcohol abuse.^45^ Moreover, due to the historical legacy of gender inequality, the health‐related consequences of gender inequality strongly affect women, especially poor women. The wage gap is common between males and females worldwide and females are often overrepresented in low‐paying jobs.^46^, ^47^ Nevertheless, the prognostic impact of this SES inequality on female patients with HCC does not seem significant in our study.

Marital status has been widely studied as an independent prognostic factor for survival in HCC patients and mainly in the form of financial and emotional support.^48^ In this study, we compared the survival of different marital status and eventually found that patients who were married or with domestic partners had the longest survivals. In contrast, the survival of patients who were single or who had unknown or another marital status (divorced, separated, and widowed) decreased in order. Married patients were more likely to comply with timely diagnosis and treatment at more highly recognized centers and accept more aggressive treatment due to patients obtaining health insurance and financial support from their spouses to cover fees of cancer treatment.^29^, ^31^, ^49^ Moreover, due to financial burden, HCC patients may increase the risk of depressive symptoms and lead to immuno‐suppression and tumor progression,^27^, ^50^ while the emotion pillar received from spouses is beneficial for patients to decrease this risk.^29^ Noteworthy, divorced, separated, and widowed patients may have clinically significant distress than other patients, leading to poorer outcomes in this population.^51^ Insurance is an important sign of SES as Medicaid insurance is an income‐based insurance program, and private insurance is high‐cost.^52^ Accordingly, insurance coverage is higher for those with higher levels of family income, whereas those with lower income rely mainly on government‐provided insurance.^53^ The difference between Medicaid insurance and private insurance comes into play when certain treatments may not be covered by insurance, or when the patient has to pay too much for some of them. It is because the latter often has the ability to cover these costs. HCC patients with insurance are more likely to get diagnosis at an earlier stage of disease and timely access to care to improve prognosis while patients without insurance may have delayed treatment.^54^ Furthermore, patients with private insurance can have earlier positions on the wait‐list of liver transplant for patients with HCC.^55^ However, in patients with advanced HCC, the effect of clinical intervention is small, making insurance disparities less pronounced.^56^ Even though a small percentage of patients may be uninsured because they can afford to pay for their own care when necessary, in general patients with insurance tend to have a better prognosis due to their own better SES and insurance covering some or all of their treatment costs.

Obviously, income can directly reflect individual’s SES, which is closely related to the diagnosis of disease and patients’ compliance to treatments. Also, income can indirectly influence patients’ marriage, insurance, and residence. County‐level education indirectly represents the level of educational development of a region. High‐educated patients are more likely to accept early screening and follow‐up treatment, while less‐educated patients tend to live in environment with low income and have lower probabilities of getting married, thereby affecting them to choose unhealthy lifestyles, such as smoking, abusing alcohol, and engaging sexual behaviors that increase the risk of viral infections.^57^ Generally, the distance of HCC patients to liver transplant and academic cancer centers also affects patient survival rates. So, patients who live in metropolitan, where has relatively better education, economy, and medical care, have more opportunities to access medical resources.^23^ However, income, residence, and education were excluded based on the univariate analysis and multivariate analysis, while poverty rate and COLI were included in our study. This may be related to the intrinsic association of these socioeconomic factors. Similar to residence, poverty rate at county level also represents a developmental level of a region. Because of their low quality of life and bad living habits, the poor population has a high prevalence of HCC, but their diagnosis and treatment are lagging behind.^58^ Given that poor people often live in geographically proximate communities, community‐targeted interventions are particularly effective public health strategies.^58^, ^59^, ^60^ This research included COLI, and as mentioned before, the index can compare the spending in different parts of the United States and the value of COLI is >1, indicating its quality of life is above the national average. Since patients spend more on necessities of life, their quality of life is naturally higher, COLI can reflect their SES. Although this study identified the COLI as an independent factor for the prognosis of the HCC patients, the correlation between COLI and HCC is still unclear and lack of research.

Although this research was based on a large population from SEER database, there are some limitations. First, the socioeconomic factors provided by database were lack of related details, such as quality and stability of marital condition, and the education and poverty did not reflect individual levels. Also, socioeconomic factors such as patients’ lifestyle habits (smoking, alcohol consumption), medical expenditures, etc., which have a more important impact on prognosis were not provided. Moreover, SEER database is lack of some important clinicopathological factors, such as adjuvant therapy, comorbidities, and recurrence.

## 5 CONCLUSION

In summary, this study analyzed the clinicopathological and socioeconomic factors and found out that age, race, sex, COLI, insurance, marital status, and poverty rate were identified as independent prognostic factors for HCC patients, and nomograms for CSS and OS for HCC patients were constructed with good predictive power. Also, this research analyzed the impacts of these factors on SES individually, reflecting the fact that socioeconomic inequalities in survival remain a serious public health problem for a health care system based on equity.^61^ Currently, national cancer policies showed weaker impact on the socioeconomic inequalities in cancer survival while annual cancer services spending still increased.^29^ Therefore, targeted social support and interventions for low SES patients may be more effective in improving prognosis.

## CONFLICT OF INTEREST

None declared.

## Supporting information


**FIGURE S1**.

**TABLE S1**.

## Data Availability

The data we used in this study can be downloaded from the SEER (Surveillance, Epidemiology, and End Results Program, 1975–2016) database.
