# Supplementary material for: Association between socioeconomic status and survival in patients with hepatocellular carcinoma
Source: Cancer Med. 2021 Aug 20;10(20):7347–59. doi: 10.1002/cam4.4223 (PMC8525159; doi:10.1002/cam4.4223)
Supplement: Supplementary file 1 — FIGURE S1. [file CAM4-10-7347-s002.pdf]

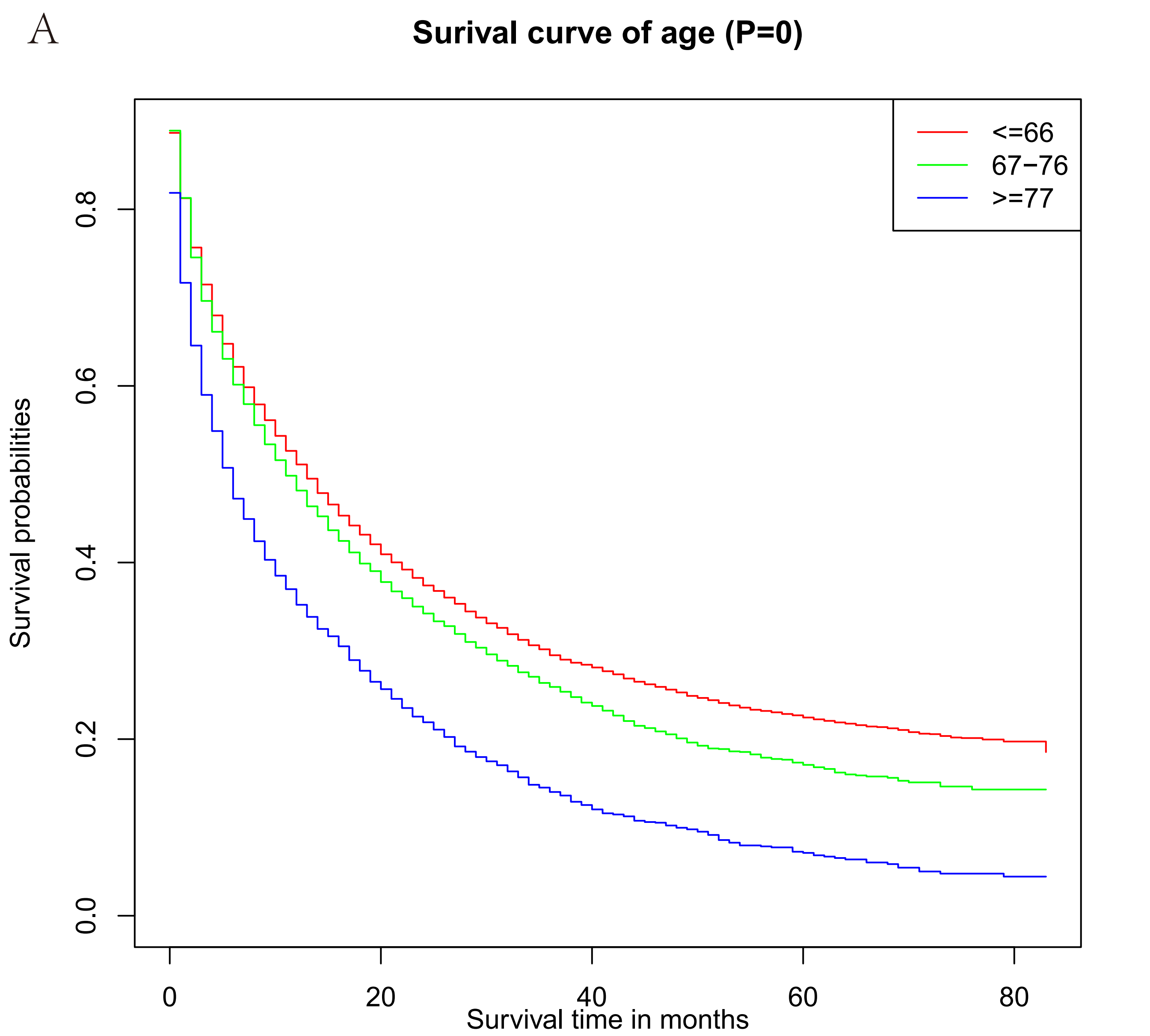
A
Surival curve of age (P=0)
<=66
67−76
>=77
Survival probabilities
0.0
0.2
0.4
0.6
0.8
0
20
40
60
80
Survival time in months

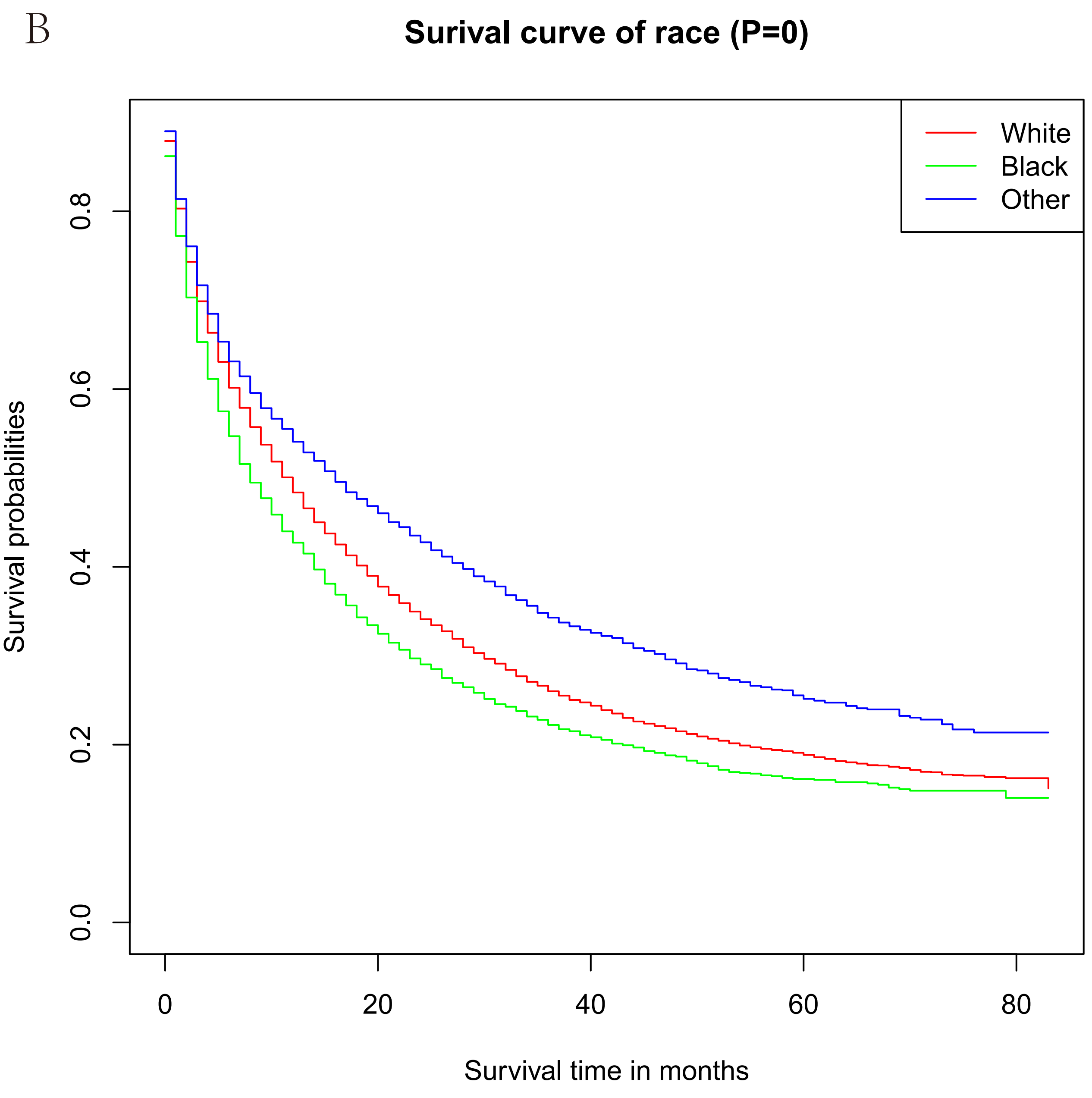
B
Surival curve of race (P=0)
White
Black
Other
Survival probabilities
0.0
0.2
0.4
0.6
0.8
0
20
40
60
80
Survival time in months

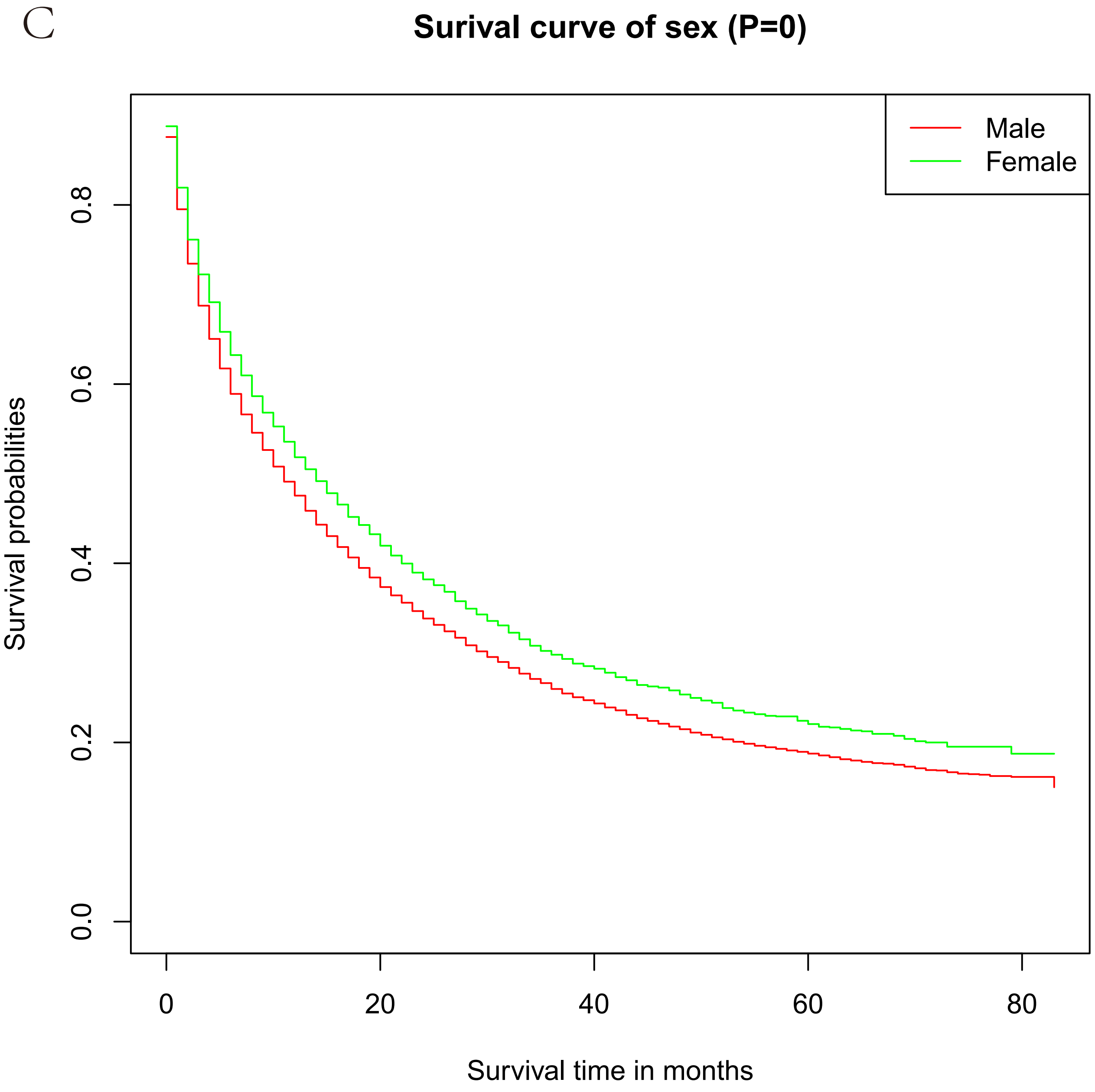
C
Surival curve of sex (P=0)
Male
Female
Survival probabilities
0.0
0.2
0.4
0.6
0.8
0
20
40
60
80
Survival time in months

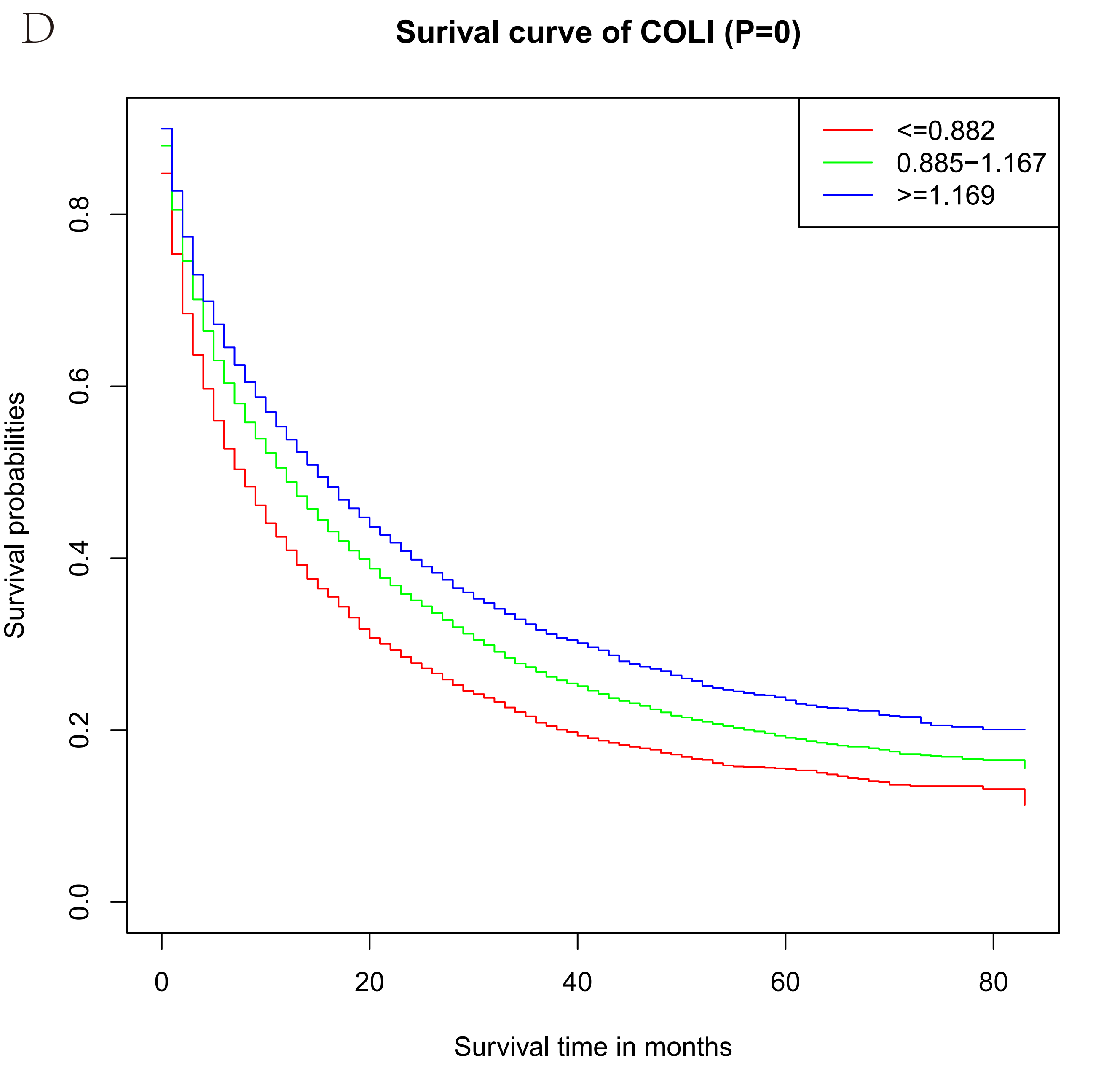

D
Surival curve of COLI (P=0)
<=0.882
0.885−1.167
>=1.169
Survival probabilities
0.0
0.2
0.4
0.6
0.8
0
20
40
60
80
Survival time in months

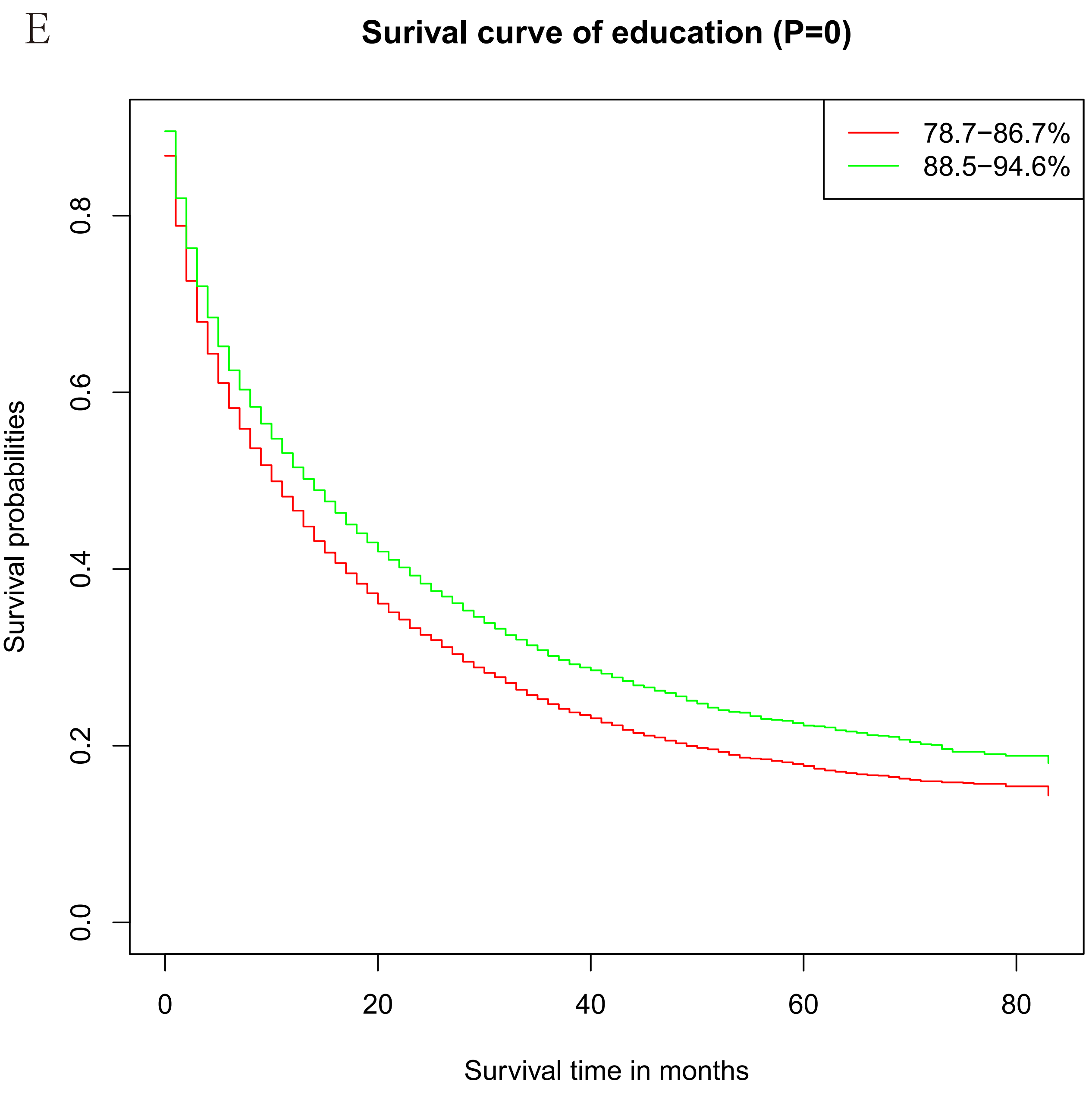
E
Surival curve of education (P=0)
78.7−86.7%
88.5−94.6%
Survival probabilities
0.0
0.2
0.4
0.6
0.8
0
20
40
60
80
Survival time in months

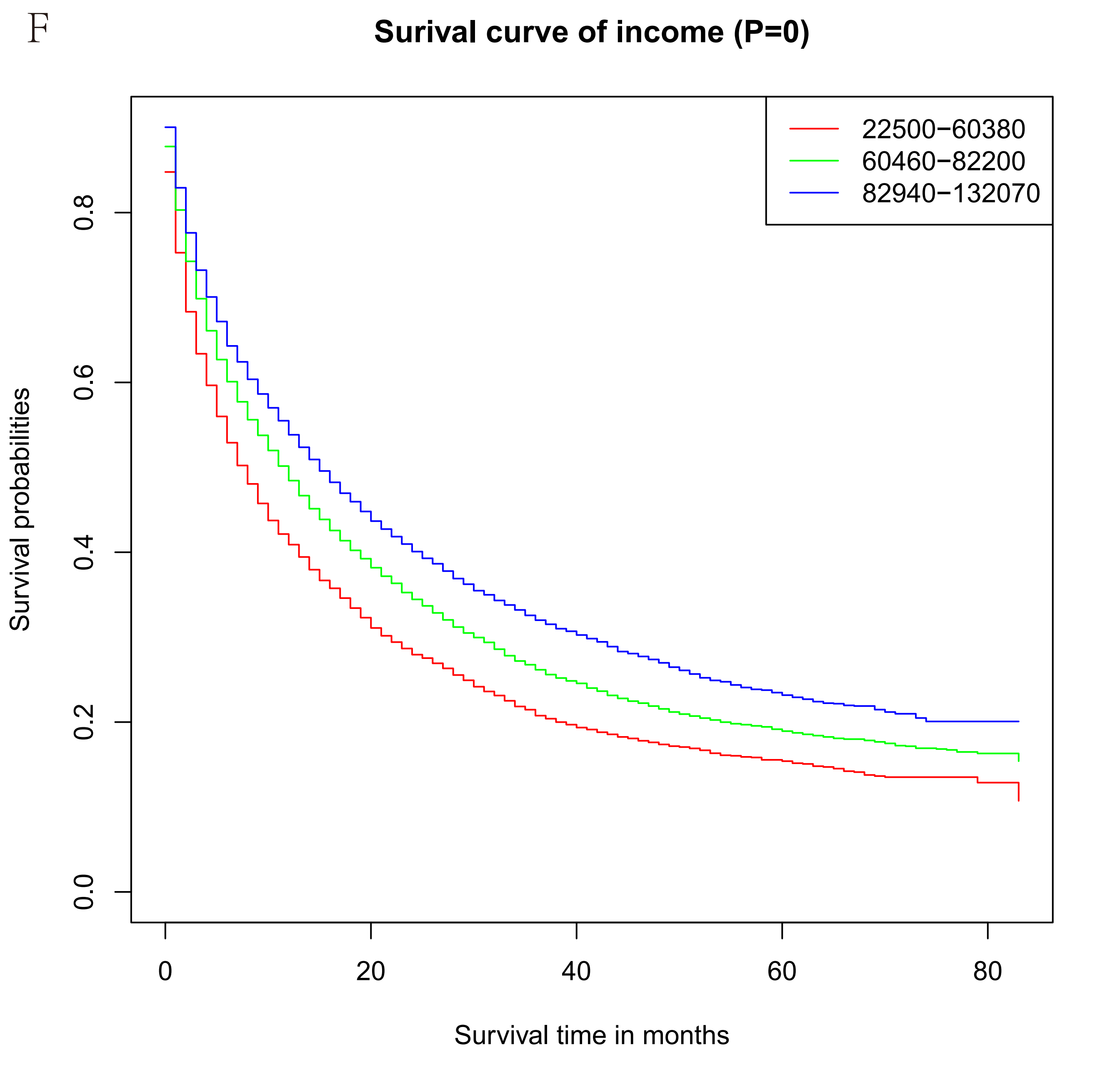
F
Surival curve of income (P=0)
22500−60380
60460−82200
82940−132070
Survival probabilities
0.0
0.2
0.4
0.6
0.8
0
20
40
60
80
Survival time in months

G

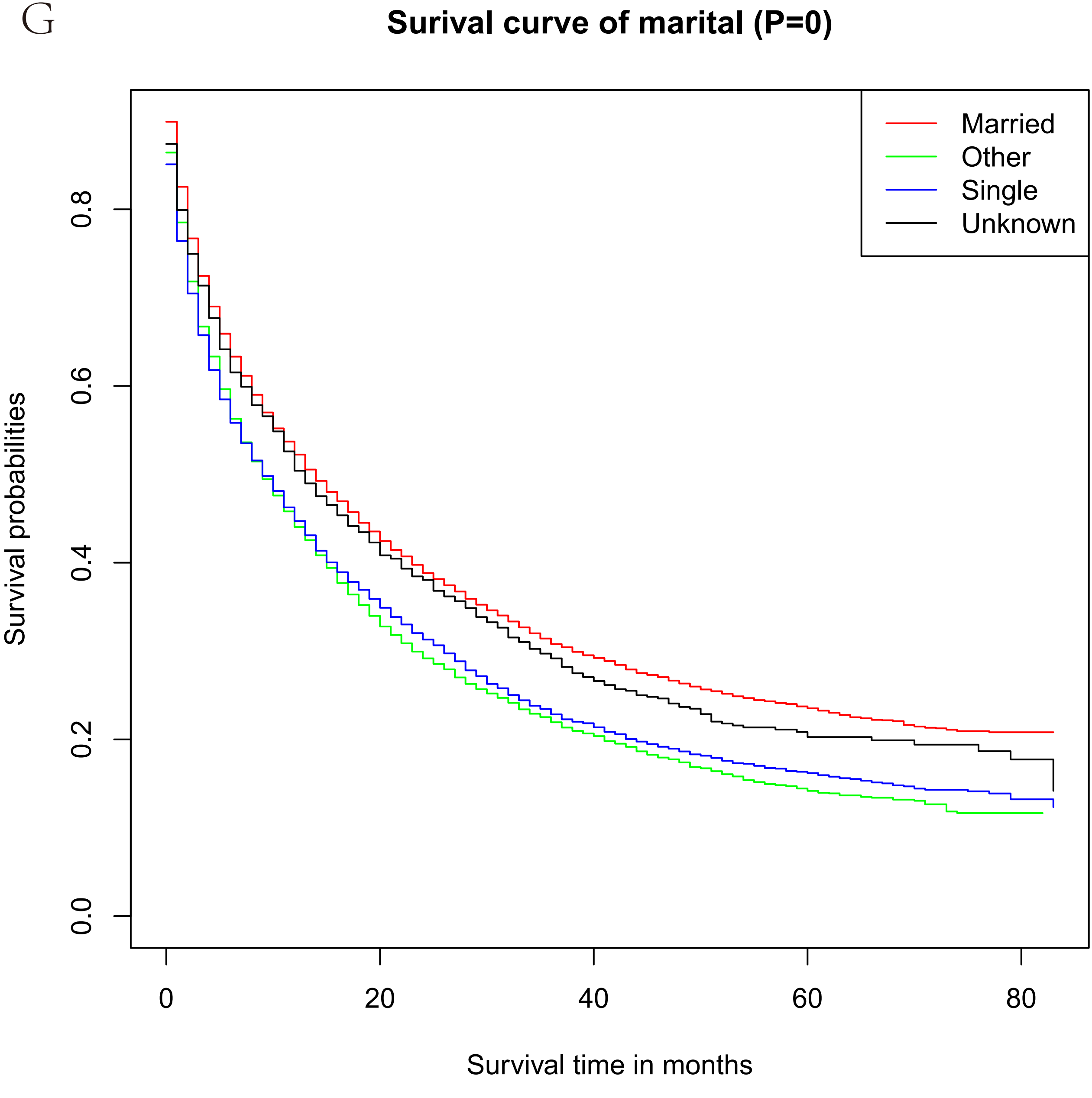

Surival curve of marital (P=0)
Married
Other
Single
Unknown
Survival probabilities
0.0
0.2
0.4
0.6
0.8
0
20
40
60
80
Survival time in months

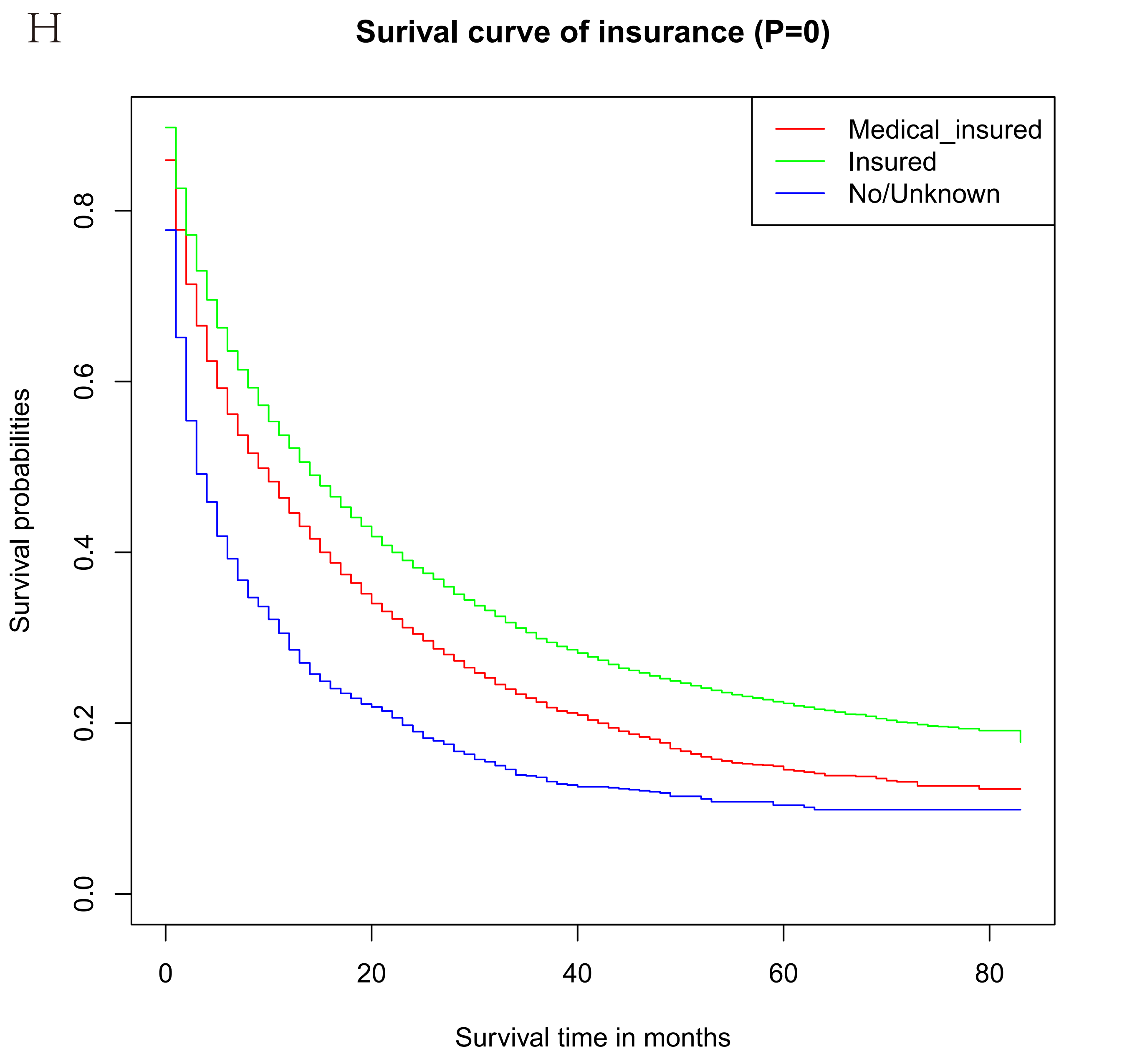
H
Surival curve of insurance (P=0)
Medical_insured
Insured
No/Unknown
Survival probabilities
0.0
0.2
0.4
0.6
0.8
0
20
40
60
80
Survival time in months

I

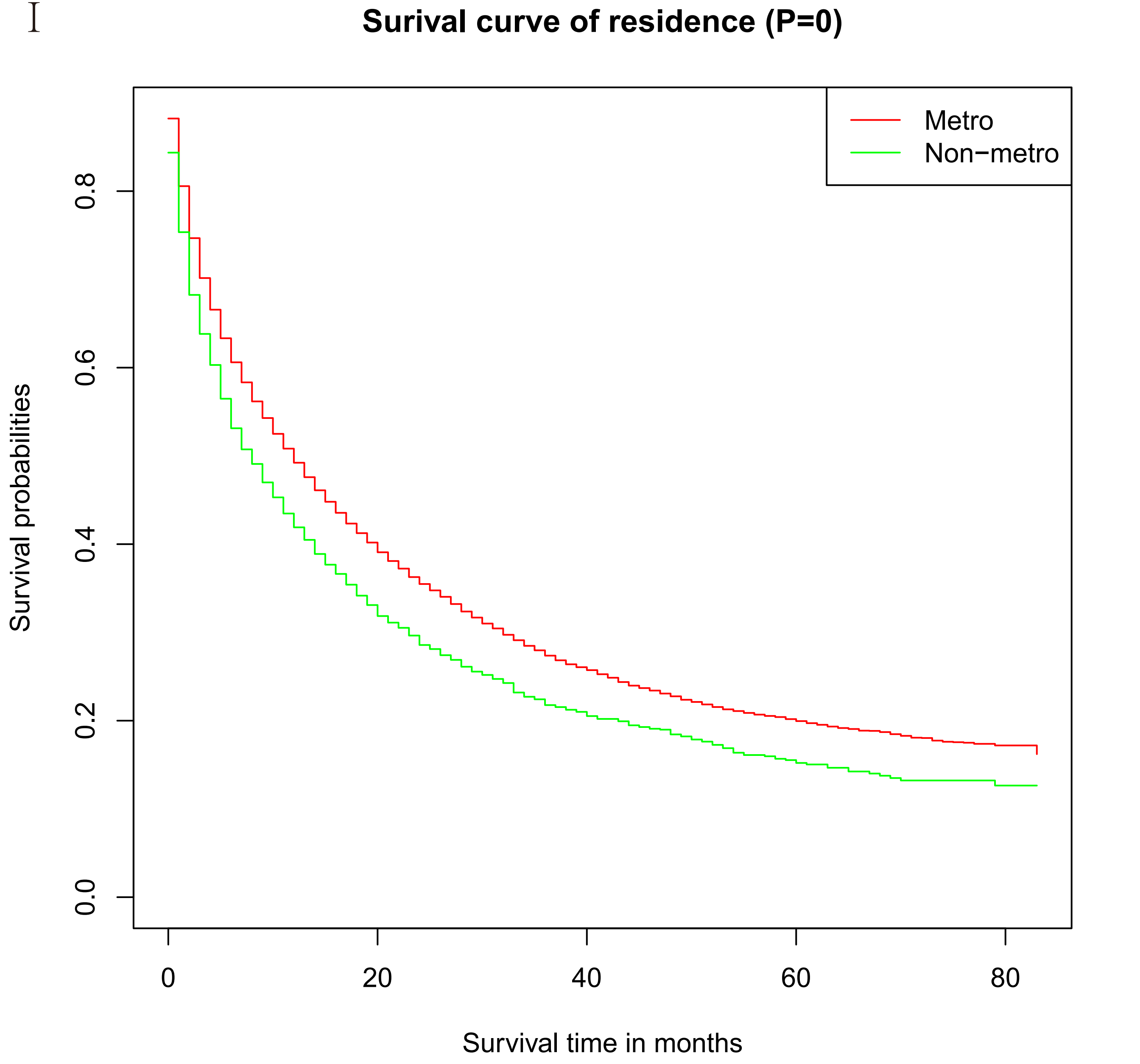

Surival curve of residence (P=0)
Metro
Non−metro
Survival probabilities
0.0
0.2
0.4
0.6
0.8
0
20
40
60
80
Survival time in months

J

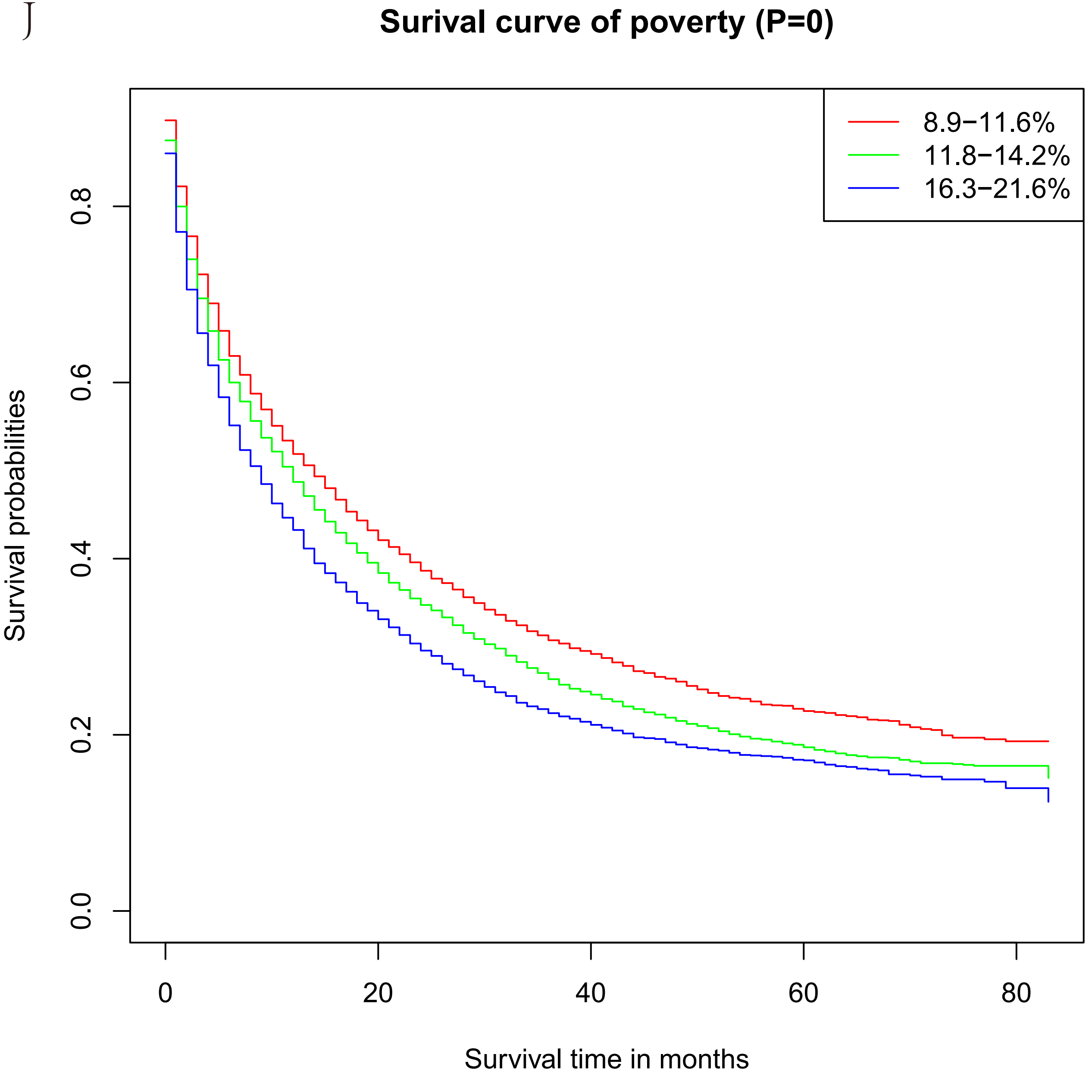

Surival curve of poverty (P=0)
8.9−11.6%
11.8−14.2%
16.3−21.6%
Survival probabilities
0.0
0.2
0.4
0.6
0.8
0
20
40
60
80
Survival time in months

K

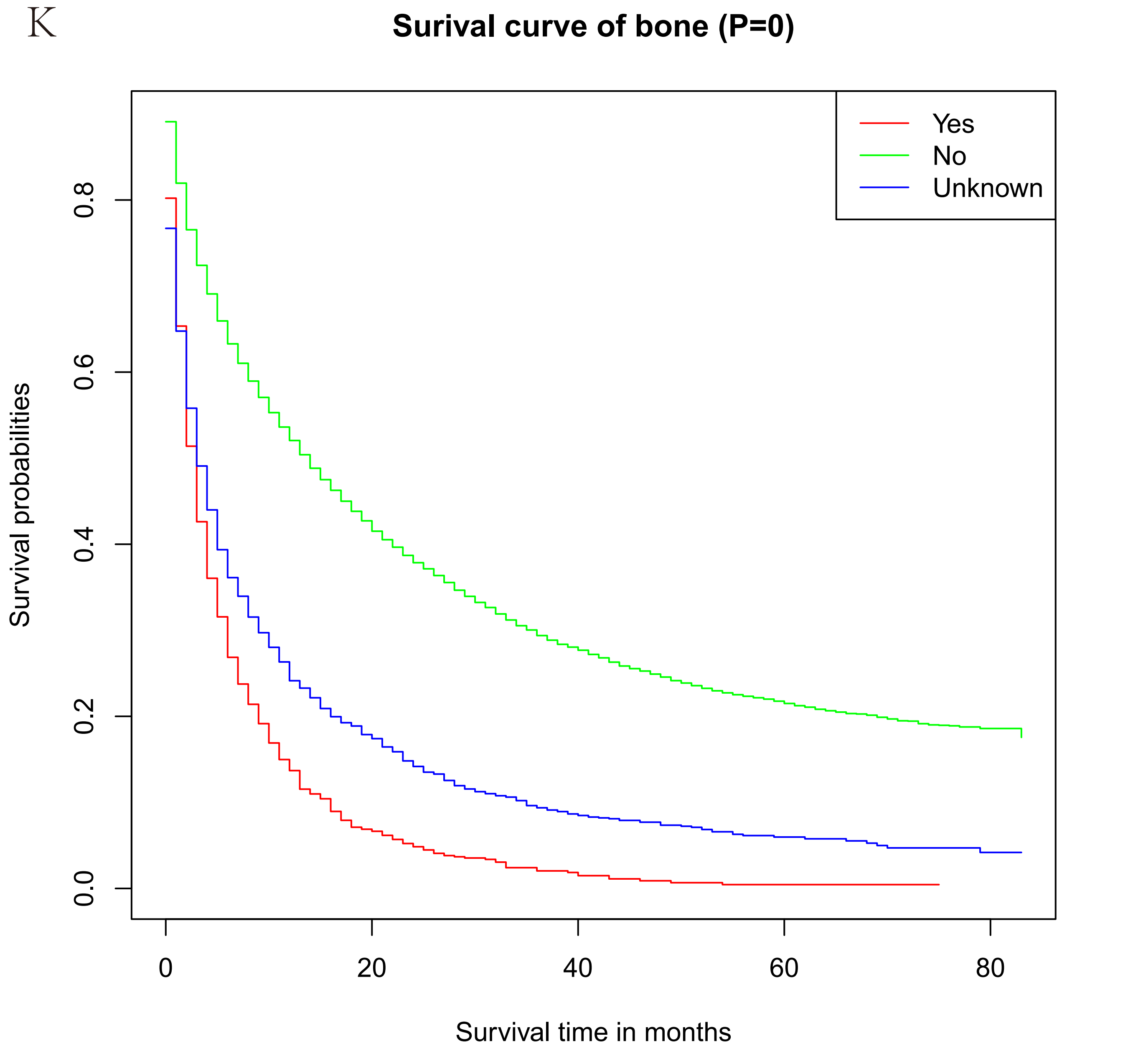
Surival curve of bone (P=0)
Yes
No
Unknown
Survival probabilities
0.0
0.2
0.4
0.6
0.8
0
20
40
60
80
Survival time in months

L

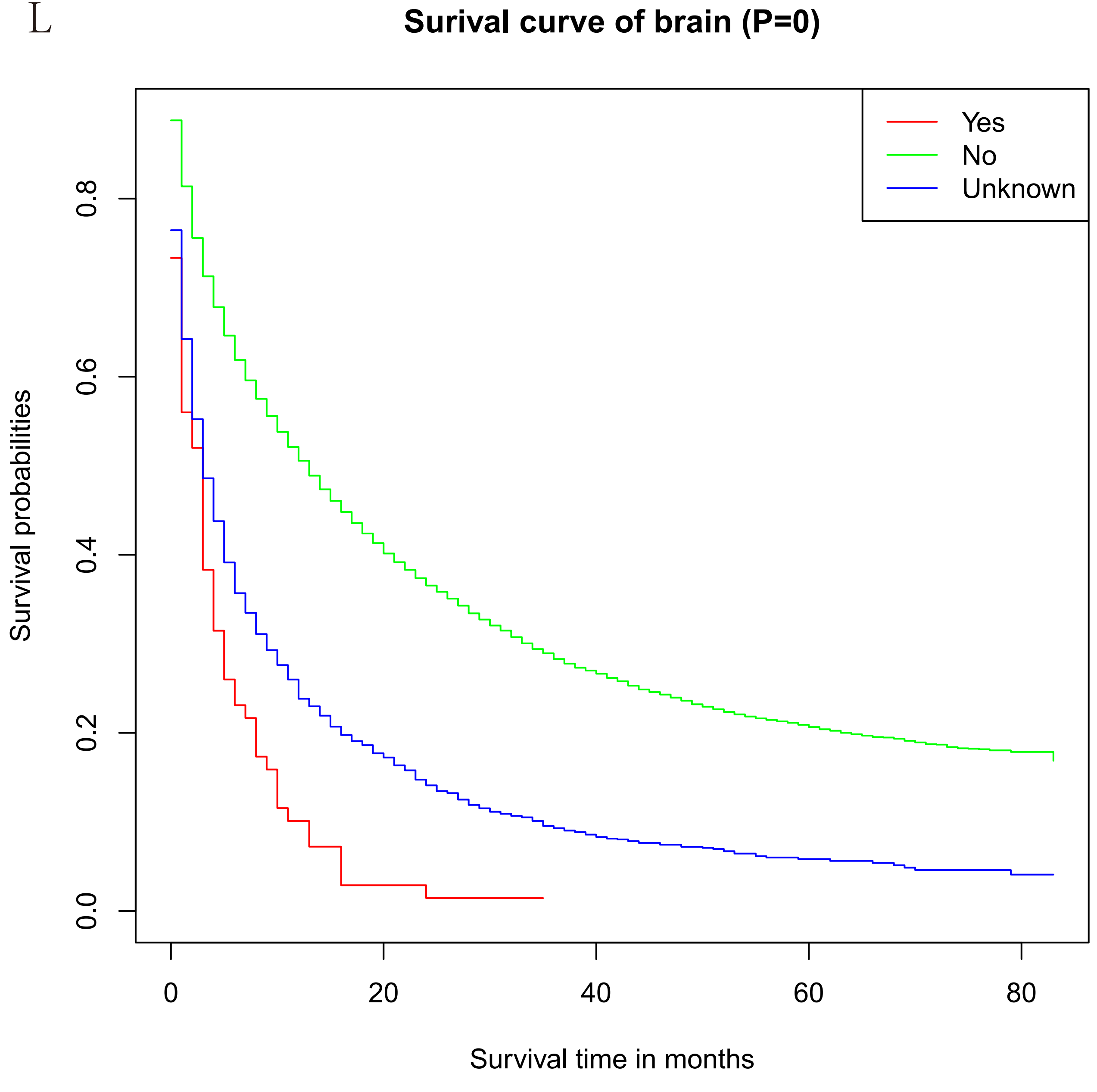
Surival curve of brain (P=0)
Yes
No
Unknown
Survival probabilities
0.0
0.2
0.4
0.6
0.8
0
20
40
60
80
Survival time in months

M

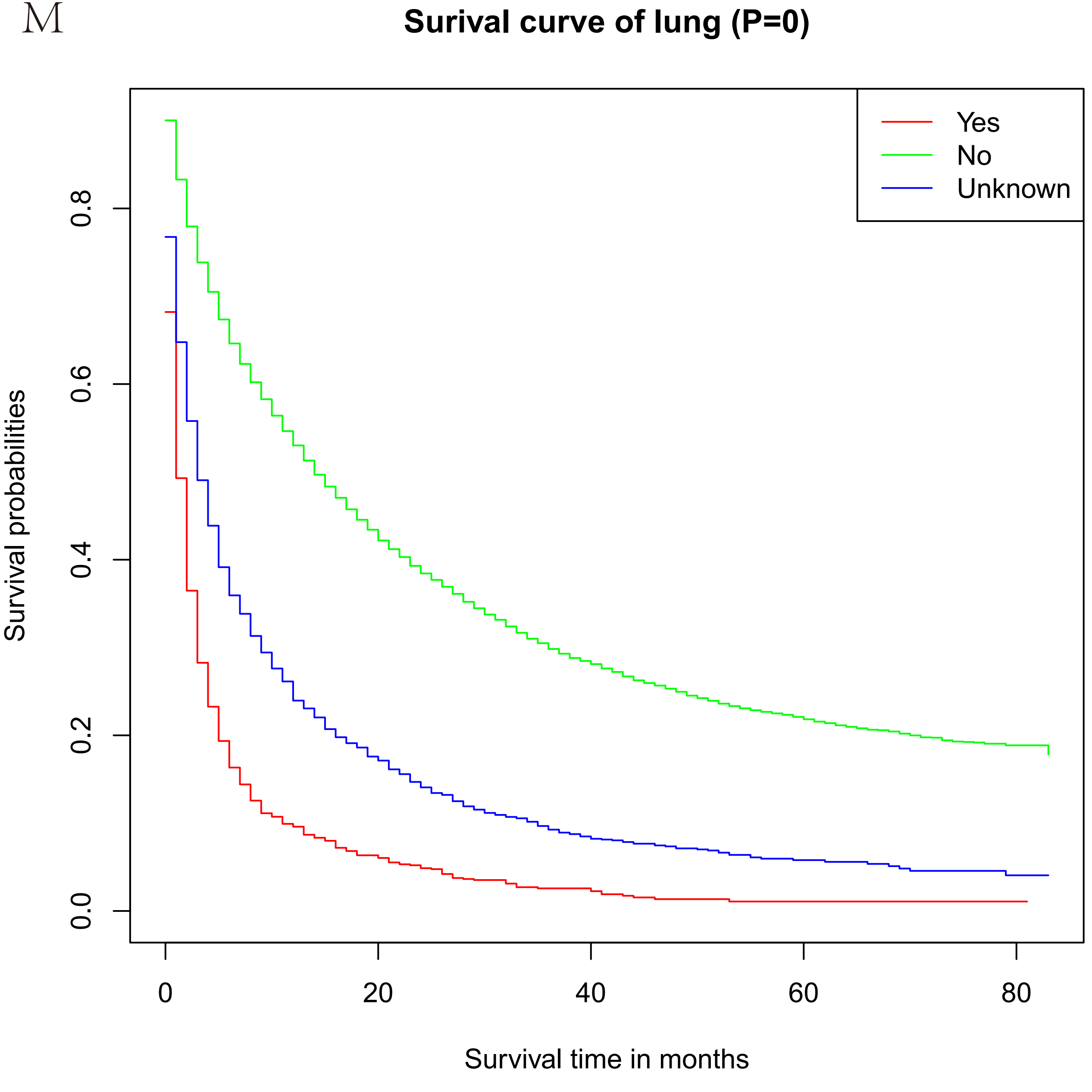

Surival curve of lung (P=0)
Yes
No
Unknown
Survival probabilities
0.0
0.2
0.4
0.6
0.8
0
20
40
60
80
Survival time in months

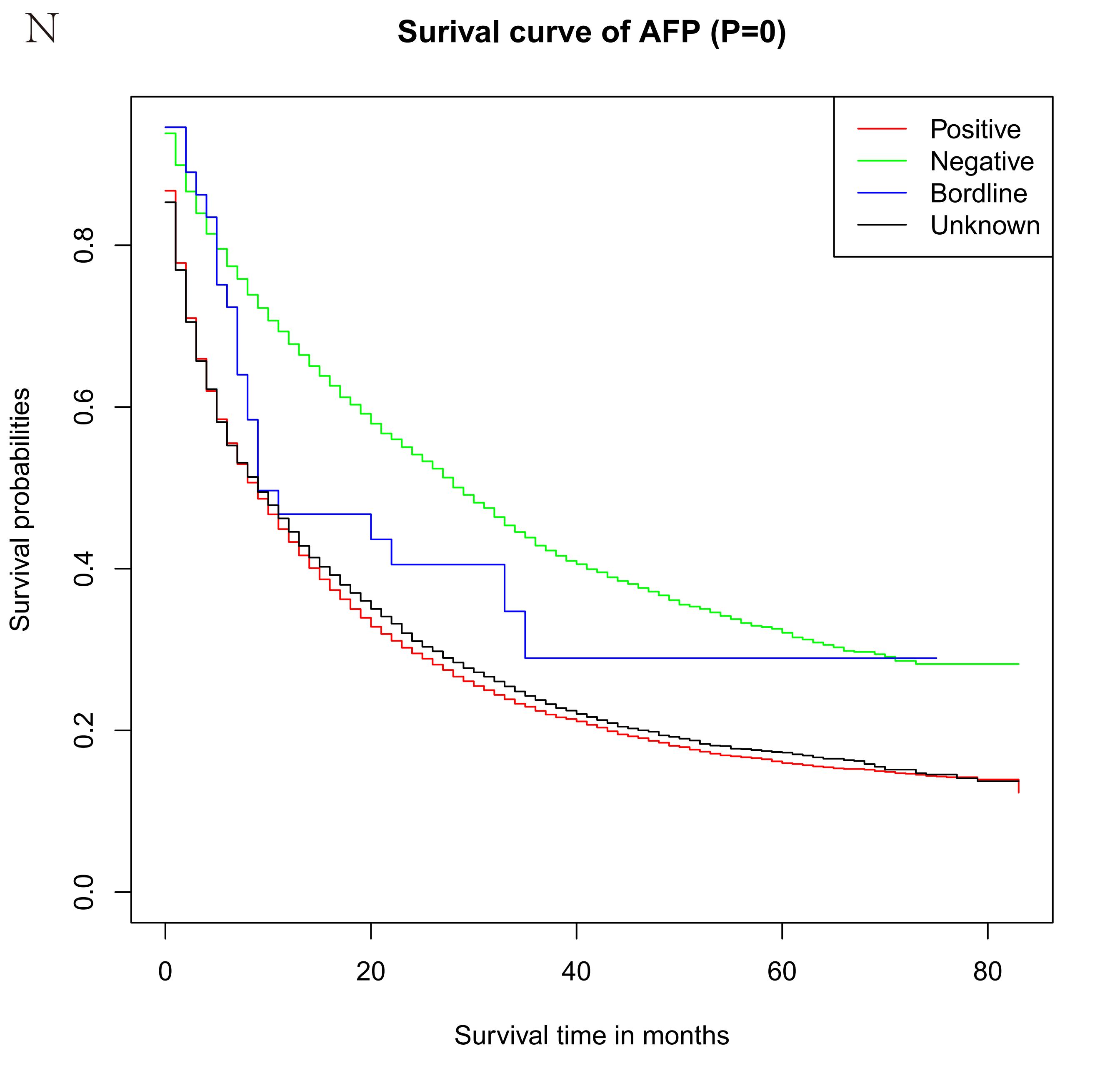
N
Surival curve of AFP (P=0)
Positive
Negative
Bordline
Unknown
Survival probabilities
0.0
0.2
0.4
0.6
0.8
0
20
40
60
80
Survival time in months

O

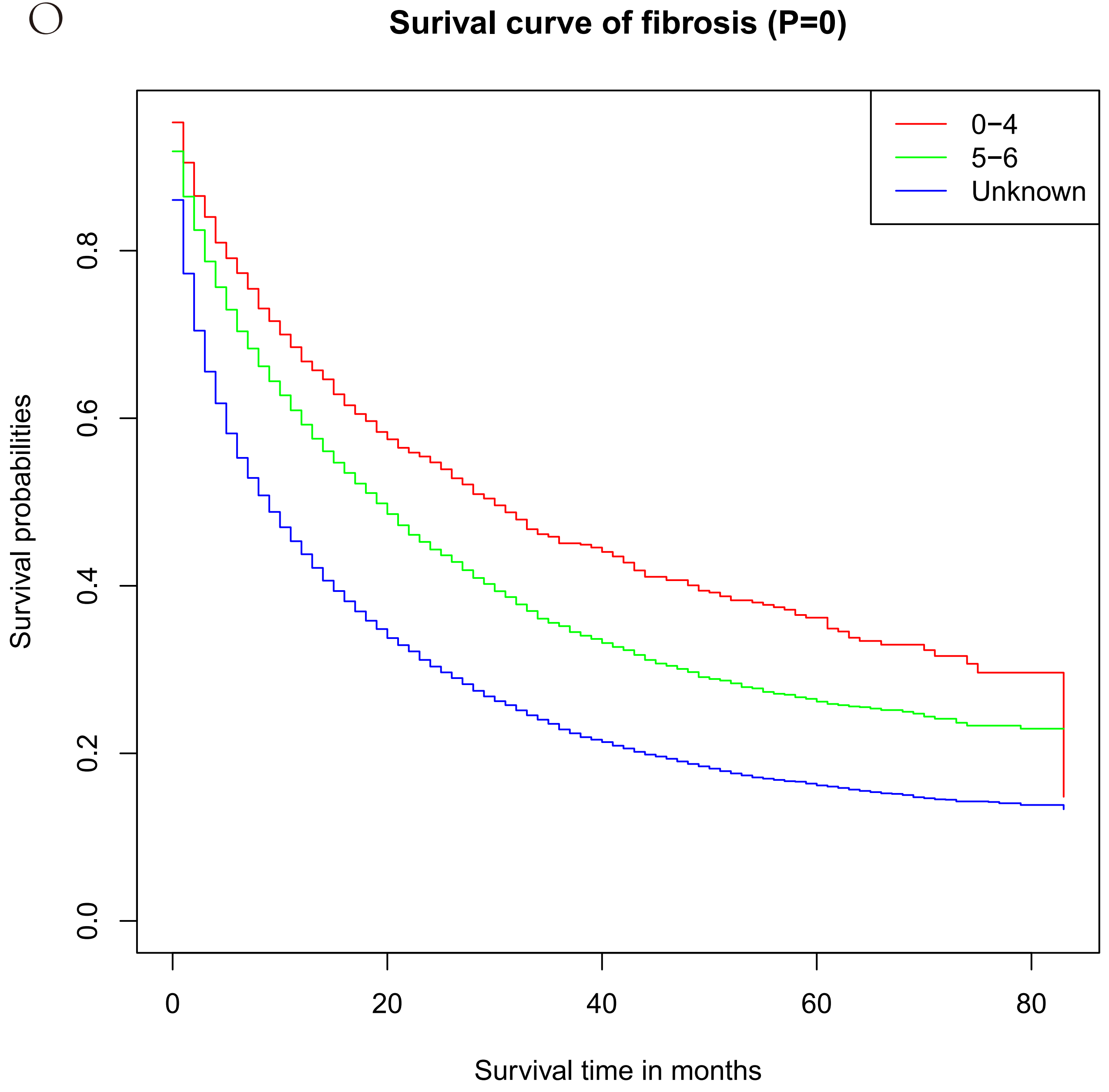
Surival curve of fibrosis (P=0)
0−4
5−6
Unknown
Survival probabilities
0.0
0.2
0.4
0.6
0.8
0
20
40
60
80
Survival time in months

P

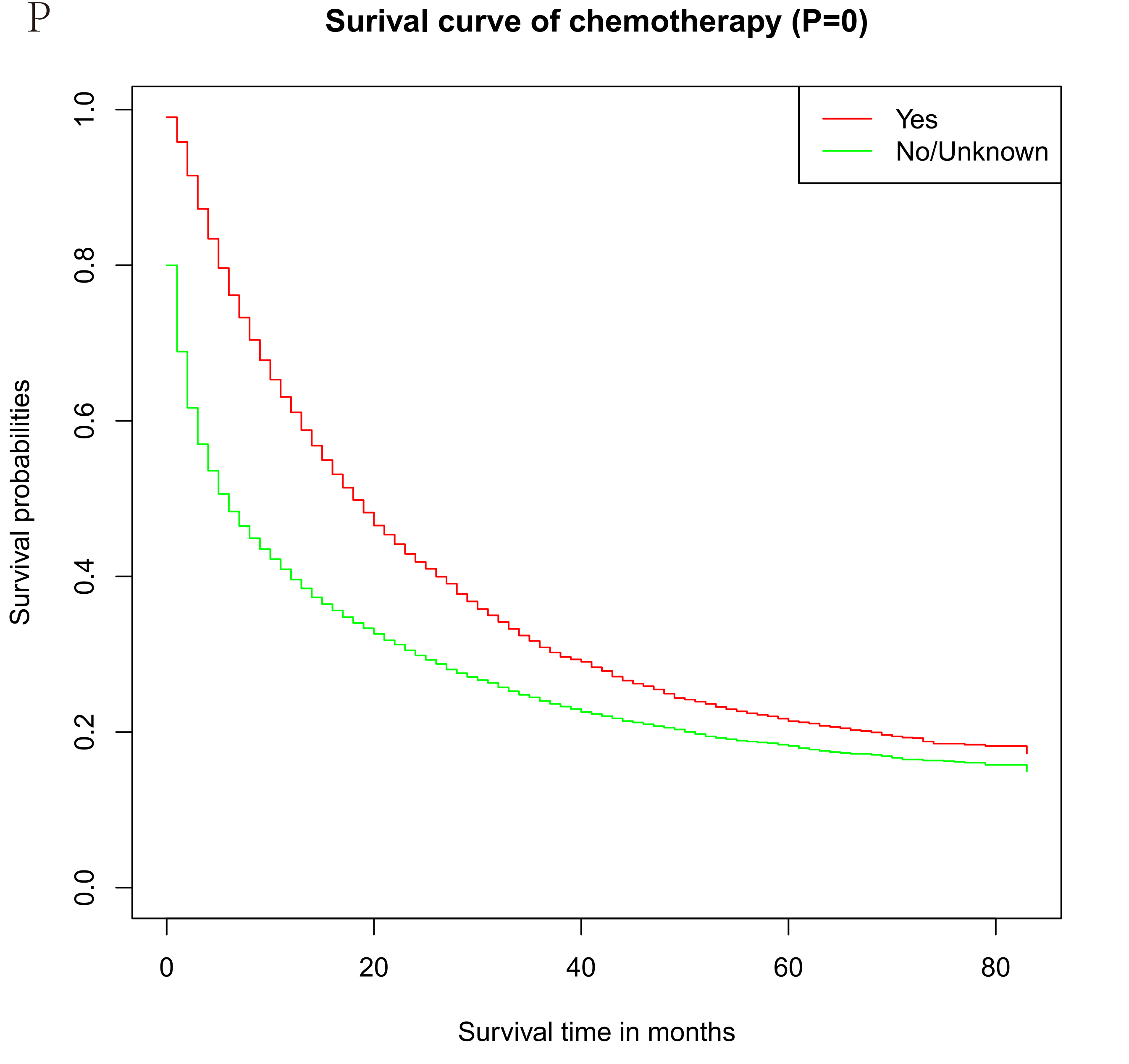

Surival curve of chemotherapy (P=0)
Yes
No/Unknown
Survival probabilities
1.0
0.8
0.6
0.4
0.2
0.0
0
20
40
60
80
Survival time in months

Q

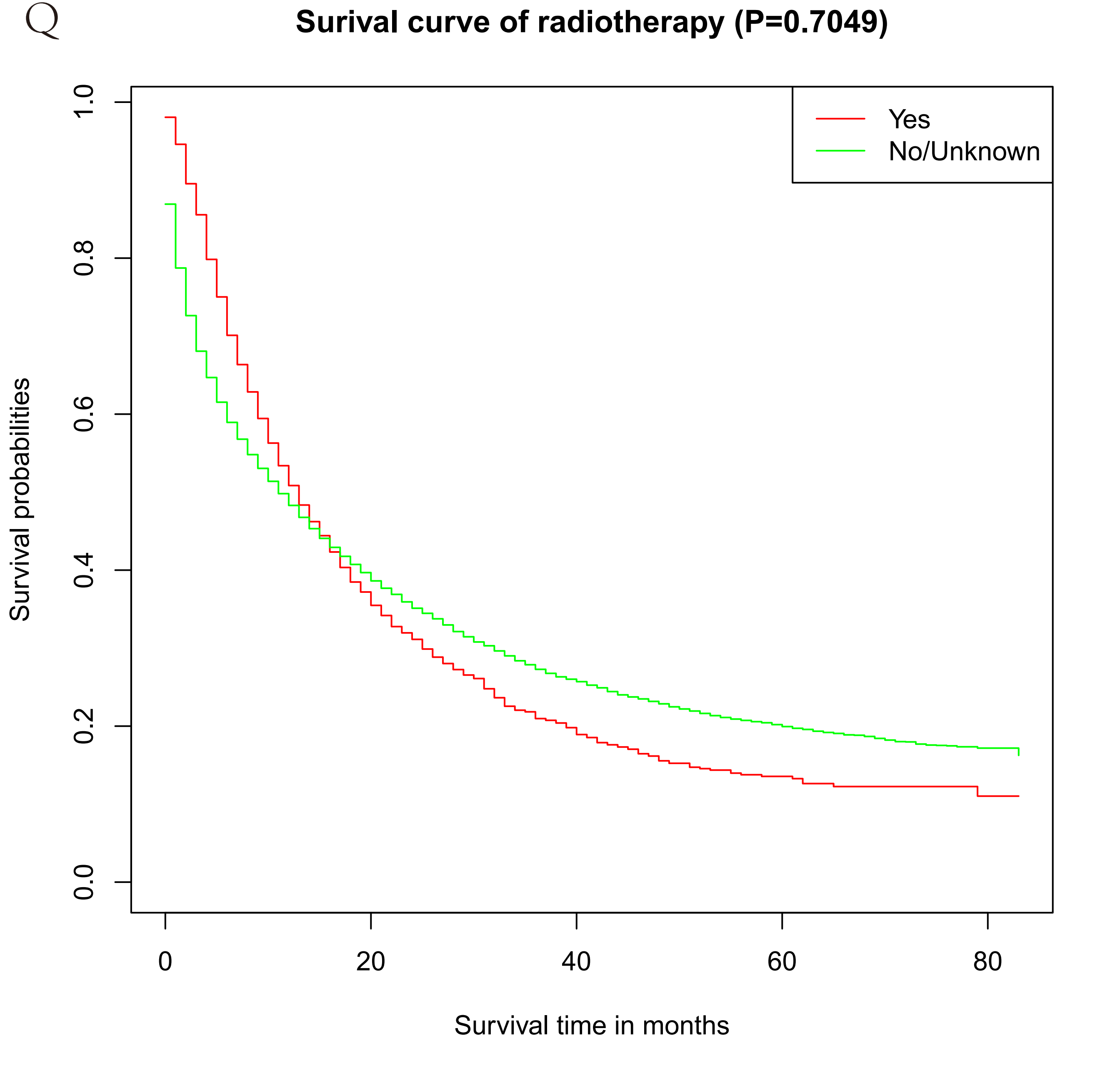
Surival curve of radiotherapy (P=0.7049)
Yes
No/Unknown
Survival probabilities
Survival time in months
0.0
0.2
0.4
0.6
0.8
1.0
0
20
40
60
80

R

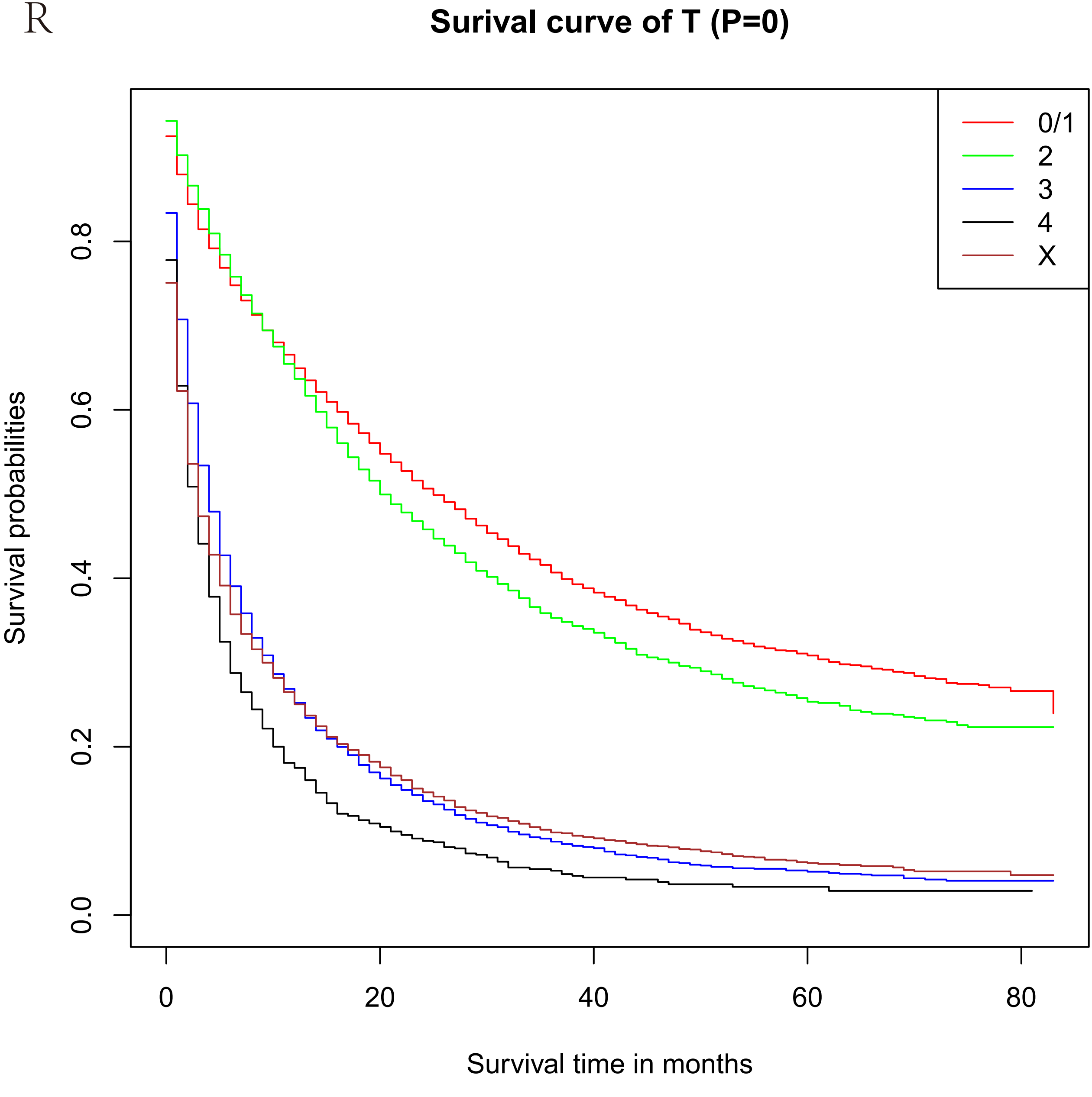

Surival curve of T (P=0)
0/1
2
3
4
X
Survival probabilities
0.0
0.2
0.4
0.6
0.8
0
20
40
60
80
Survival time in months

S

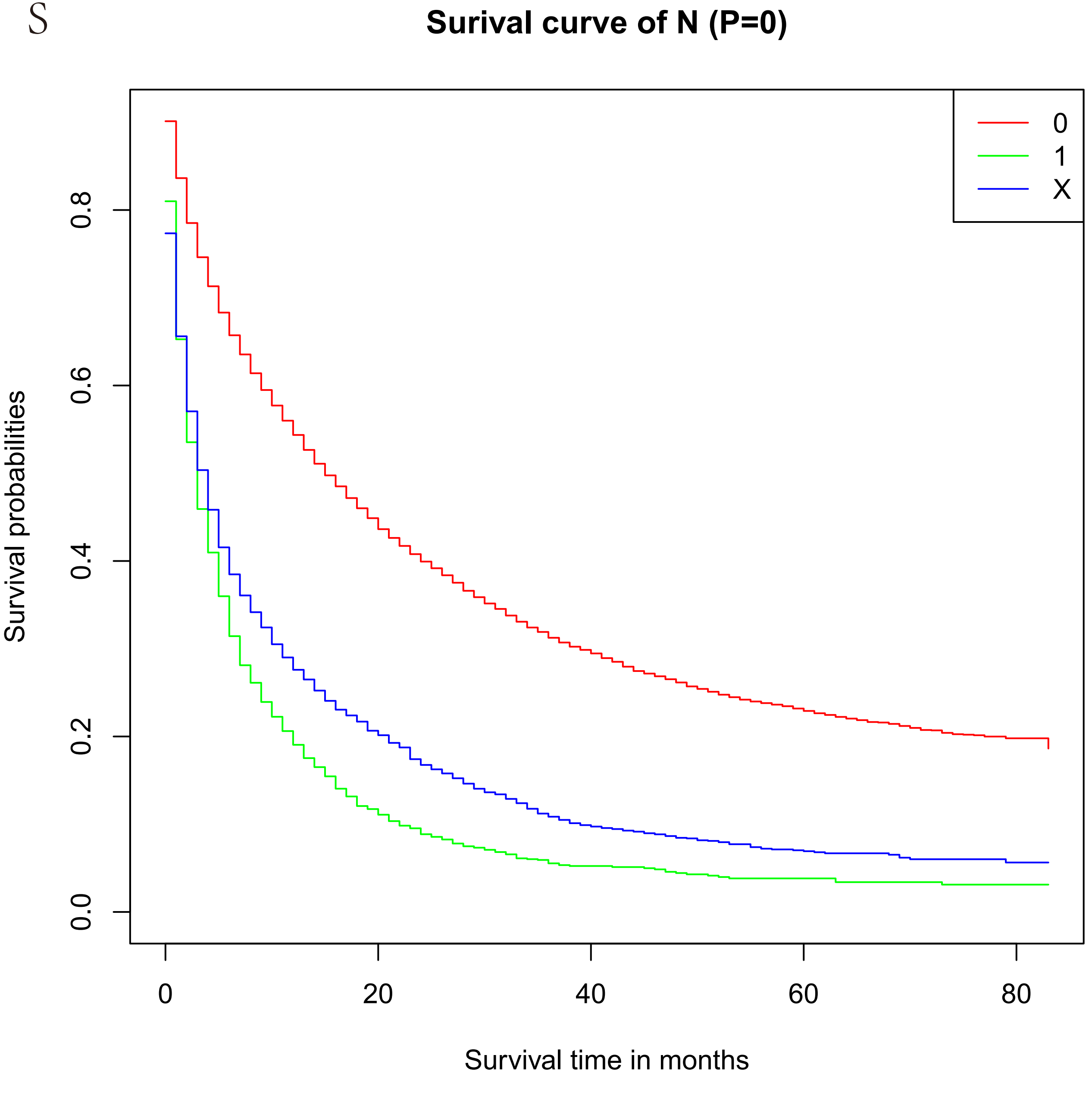
Surival curve of N (P=0)
Survival probabilities
Survival time in months
0.0
0.2
0.4
0.6
0.8
0
20
40
60
80
0
1
X

T

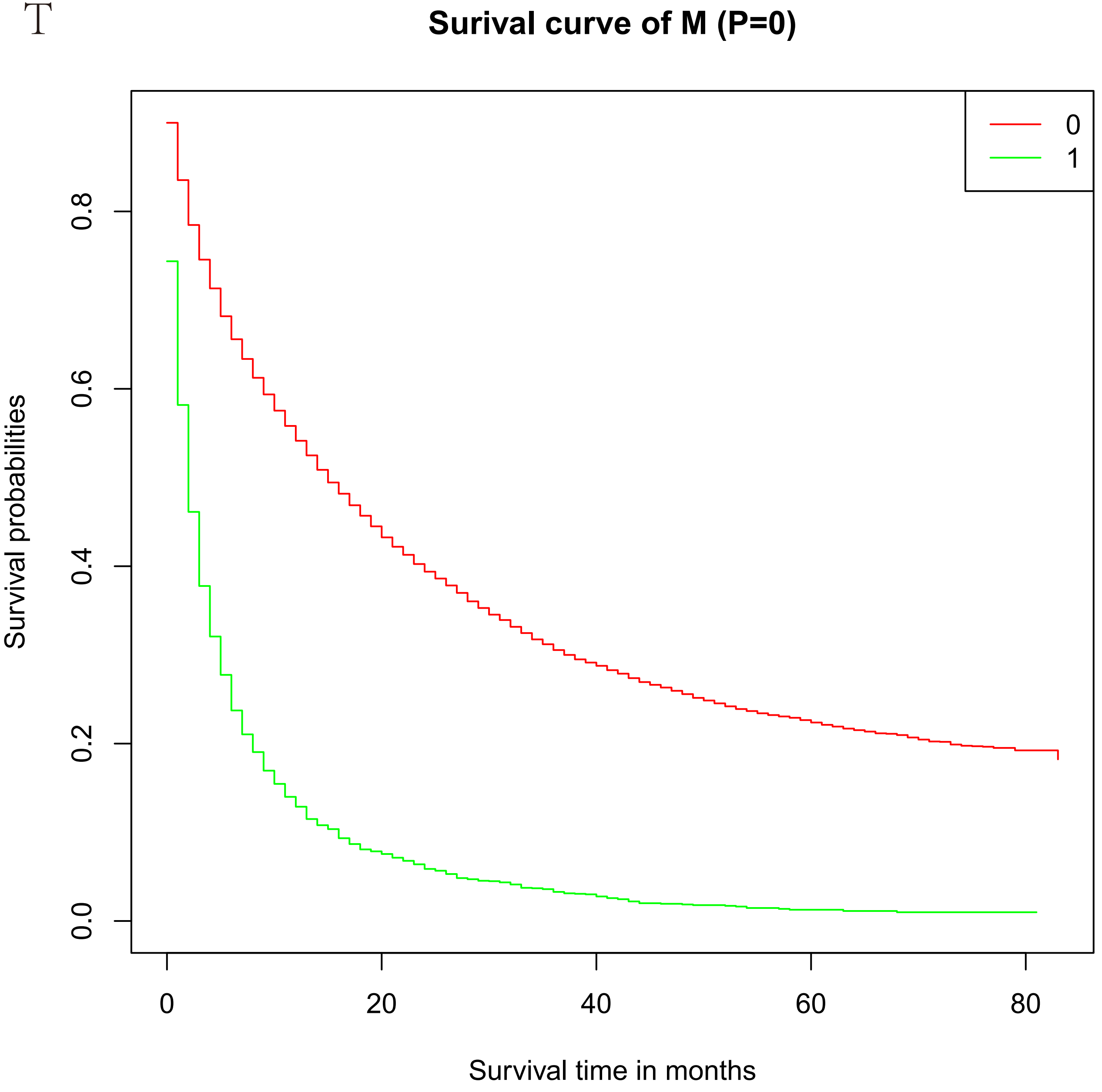

Surival curve of M (P=0)
0
1
Survival probabilities
0.0
0.2
0.4
0.6
0.8
0
20
40
60
80
Survival time in months

U

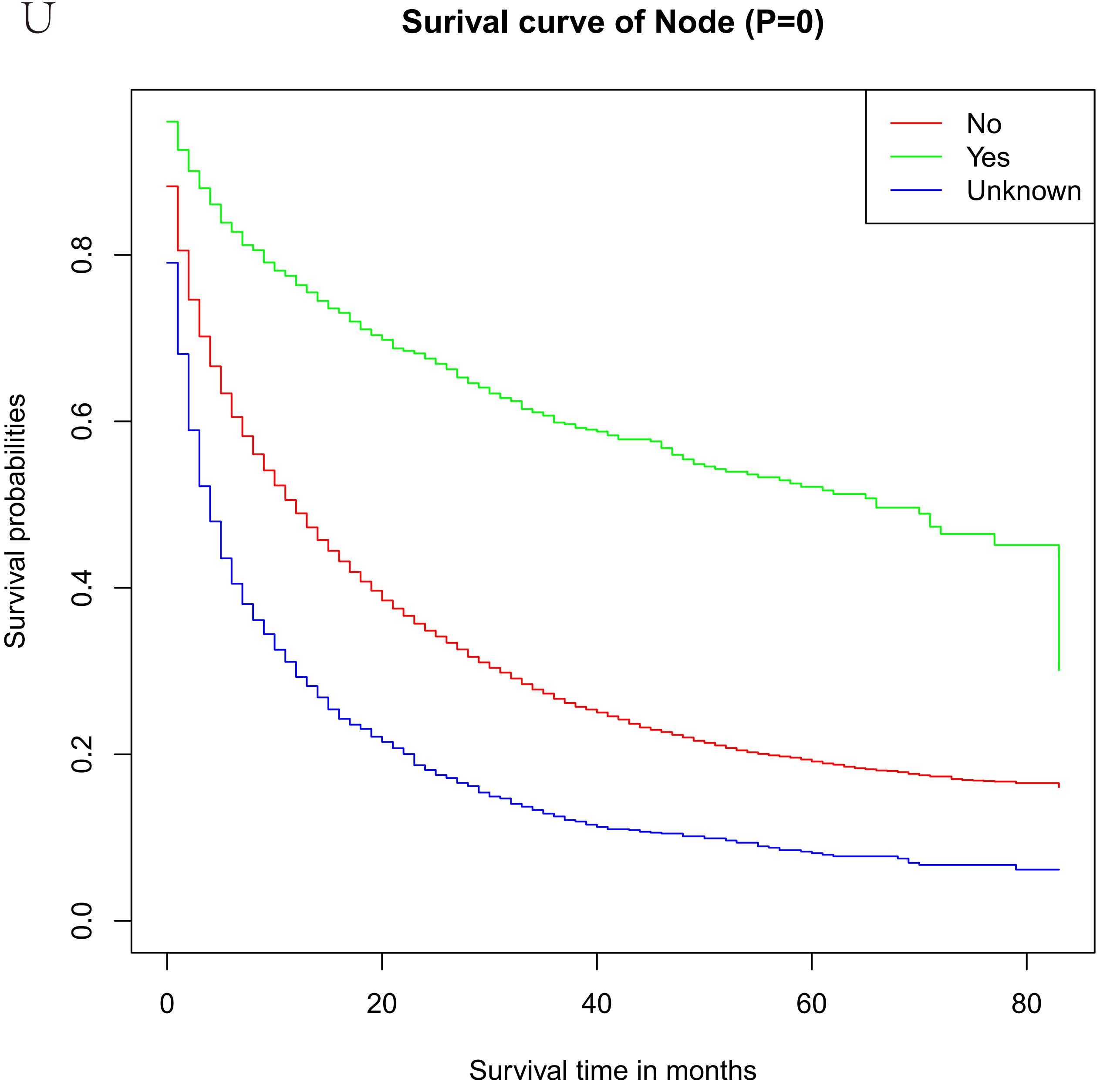
Surival curve of Node (P=0)
No
Yes
Unknown
Survival probabilities
0.0
0.2
0.4
0.6
0.8
0
20
40
60
80
Survival time in months

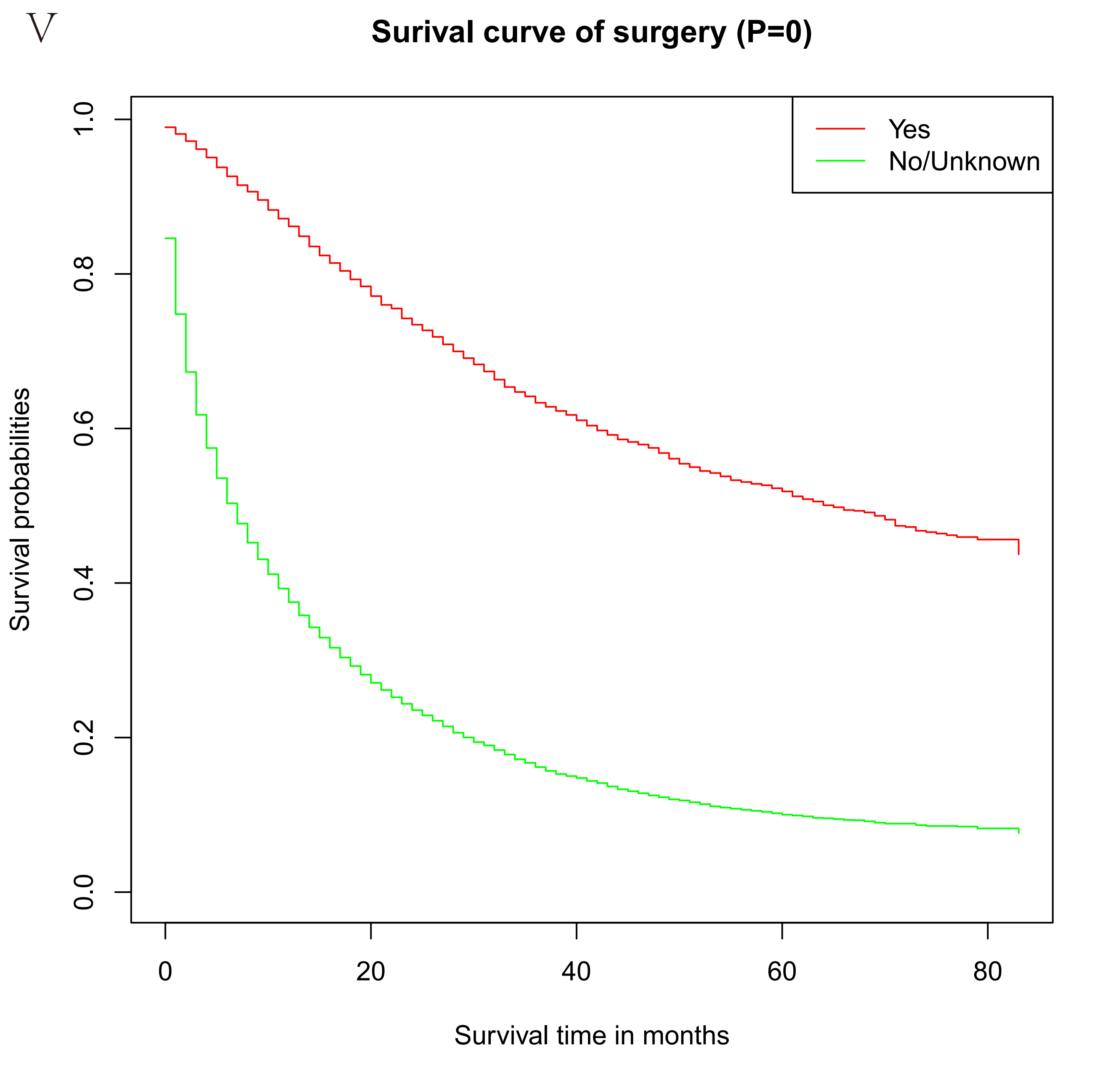
V
Surival curve of surgery (P=0)
Yes
No/Unknown
Survival probabilities
0.0
0.2
0.4
0.6
0.8
1.0
0
20
40
60
80
Survival time in months

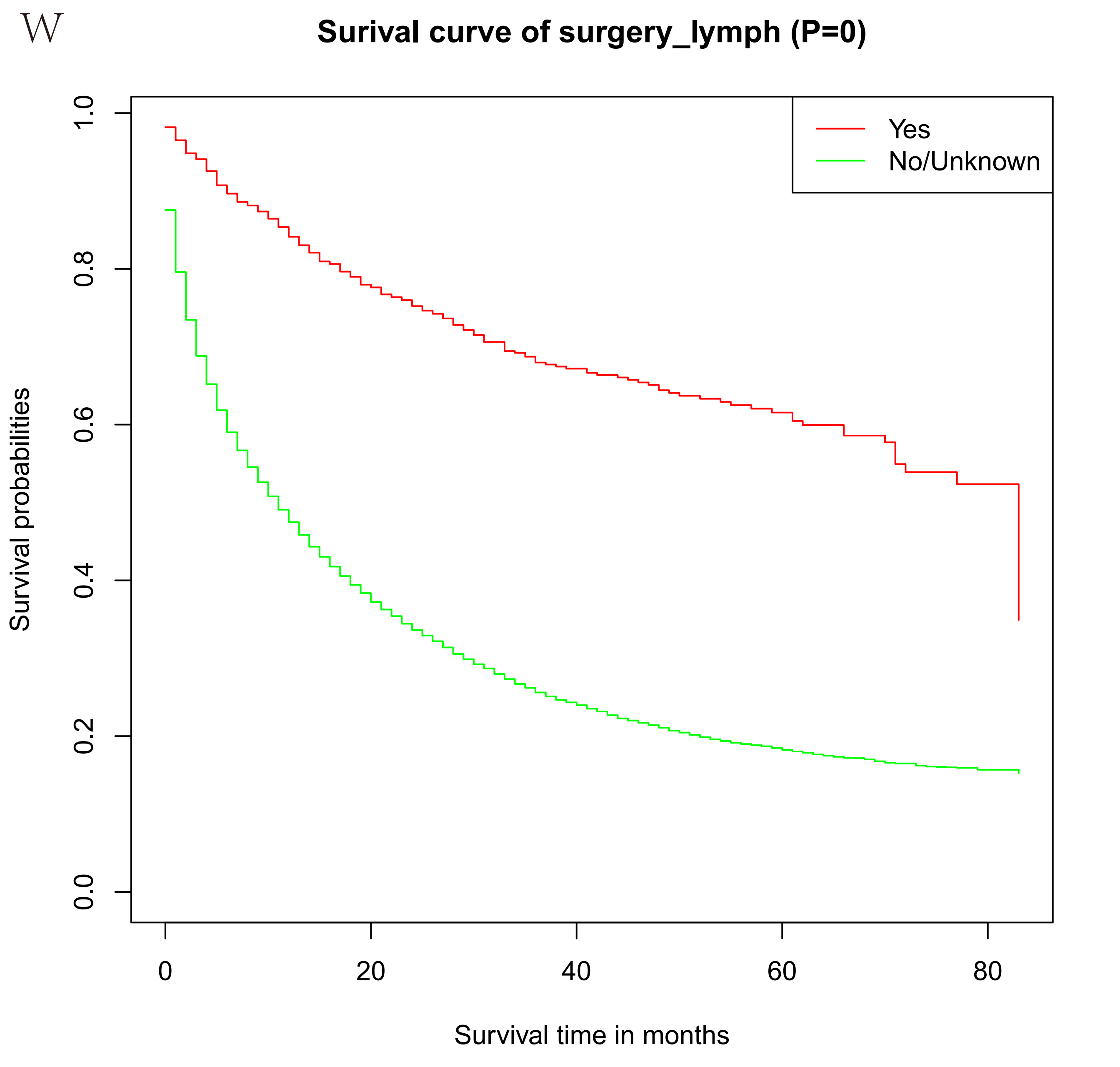
W
Surival curve of surgery_lymph (P=0)
Yes
No/Unknown
Survival probabilities
0.0
0.2
0.4
0.6
0.8
1.0
0
20
40
60
80
Survival time in months

X

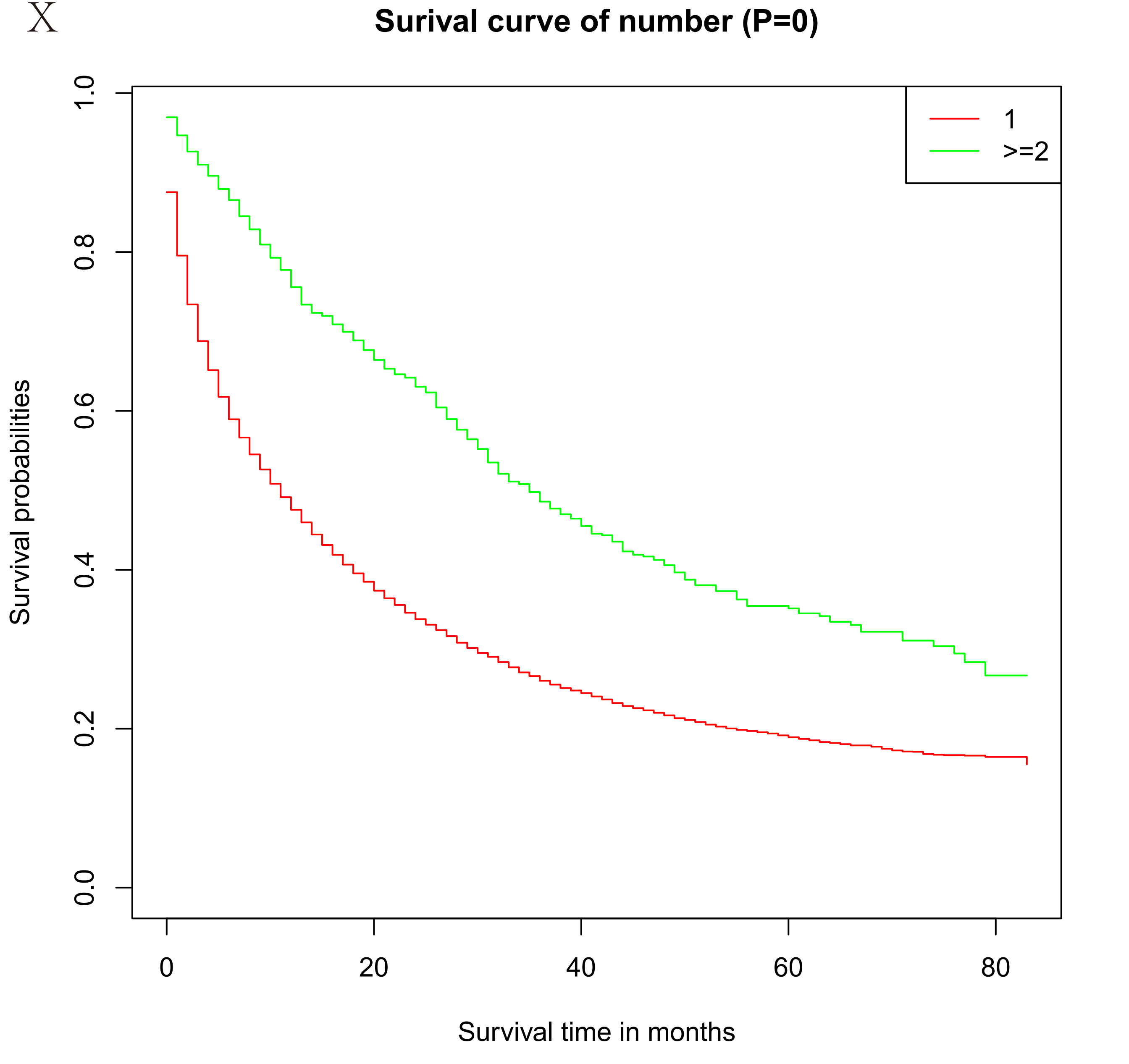

Surival curve of number (P=0)
1
>=2
Survival probabilities
Survival time in months
0.0
0.2
0.4
0.6
0.8
1.0
0
20
40
60
80

Y

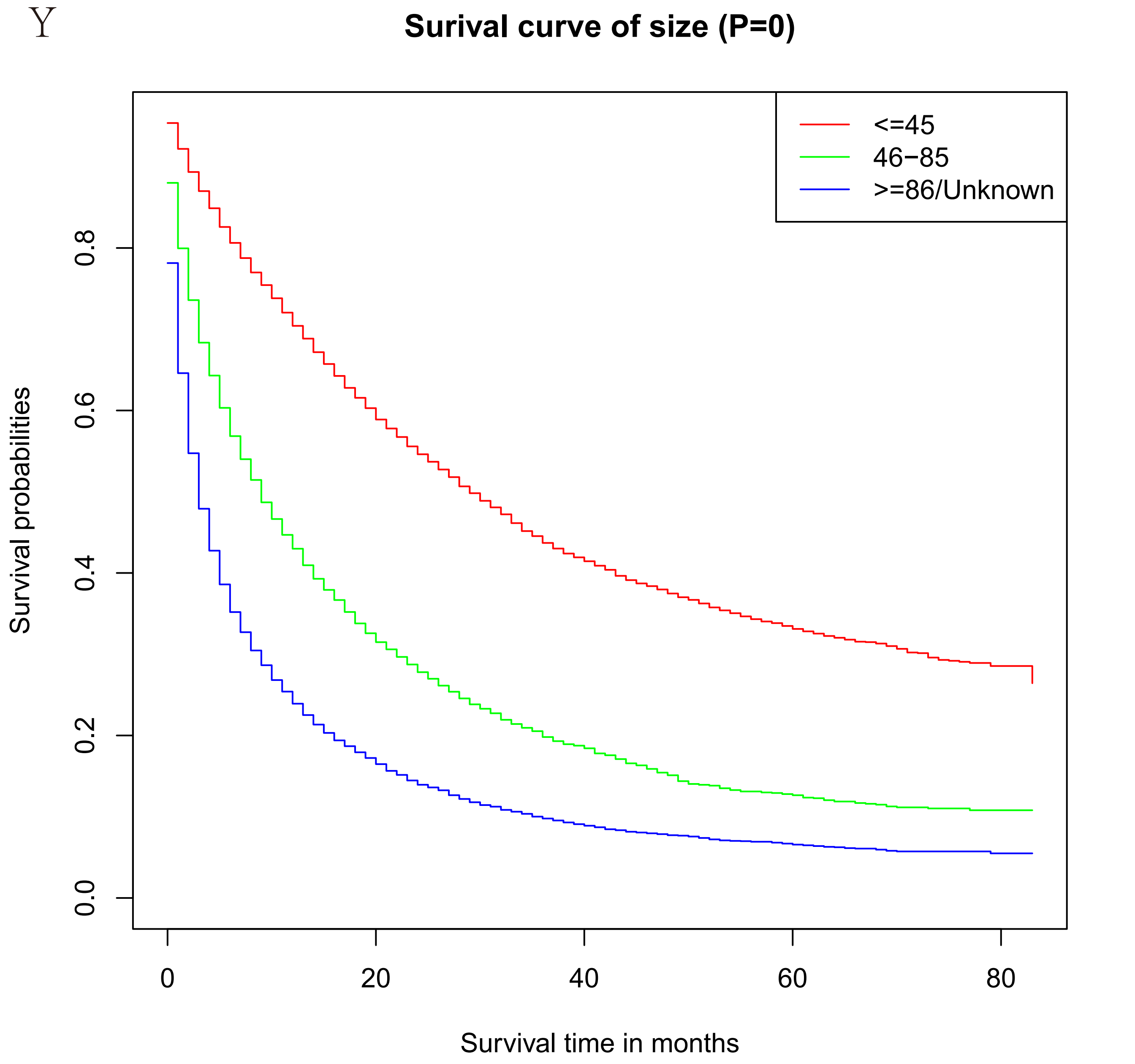
Surival curve of size (P=0)
<=45
46−85
>=86/Unknown
Survival probabilities
0.0
0.2
0.4
0.6
0.8
0
20
40
60
80
Survival time in months
