# Supplementary material for: Association between socioeconomic status and survival in patients with hepatocellular carcinoma
Source: Cancer Med. 2021 Aug 20;10(20):7347–59. doi: 10.1002/cam4.4223 (PMC8525159; doi:10.1002/cam4.4223)
Supplement: Supplementary file 2 — TABLE S1. [file CAM4-10-7347-s001.docx]

Supplemental table: IDI and NRI of the nomograms on CSS and OS

|  | Survival time | Items | Training set | Validation set |
| --- | --- | --- | --- | --- |
|  |  |  | Est (95% CI) | Est (95% CI) |
| CSS | 1-year | IDI | 0.01(0.01-0.01) *** | 0.01(0.01-0.01) *** |
|  |  | NRI | 0.16(0.11-0.19) *** | 0.09(0.02-0.16) *** |
|  | 3-year | IDI | 0.01(0.01-0.01) *** | 0.01(0.01-0.02) *** |
|  |  | NRI | 0.25(0.21-0.27) *** | 0.24(0.18-0.28) *** |
|  | 5-year | IDI | 0.02(0.01-0.02) *** | 0.02(0.01-0.02) *** |
|  |  | NRI | 0.32(0.27-0.36) *** | 0.29(0.22-0.34) *** |
| OS | 1-year | IDI | 0.01(0.01-0.01) *** | 0.01(0.01-0.01) *** |
|  |  | NRI | 0.15(0.12-0.19) *** | 0.11(0.05-0.17) *** |
|  | 3-year | IDI | 0.01(0.01-0.02) *** | 0.02(0.01-0.02) *** |
|  |  | NRI | 0.26(0.22-0.29) *** | 0.24(0.19-0.29) *** |
|  | 5-year | IDI | 0.02(0.02-0.02) *** | 0.02(0.01-0.02) *** |
|  |  | NRI | 0.33(0.28-0.37) *** | 0.28(0.23-0.35) *** |

*** p <0.001

Abbreviations: CSS, cause-specific survival; OS, overall survival; Est, Empower Stats; CI: confidence interval; IDI, integrated discrimination improvement index; NRI, category-less net reclassification index.
